# Impact of growth conditions on the abundance and diversity of cultivable bacteria recovered from *Pheronema carpenteri* and investigation of their antimicrobial potential

**DOI:** 10.1093/femsmc/xtaf016

**Published:** 2025-11-07

**Authors:** Jazmin Conway, Grant G January, Katie J Muddiman, Rosemary Dorrington, Kerry L Howell, Mathew Upton

**Affiliations:** School of Biological and Marine Sciences, University of Plymouth, Drake Circus, Plymouth PL4 8AA, United Kingdom; School of Biomedical Sciences, University of Plymouth, Drake Circus, Plymouth PL4 8AA, United Kingdom; School of Biomedical Sciences, University of Plymouth, Drake Circus, Plymouth PL4 8AA, United Kingdom; Department of Biochemistry, Microbiology and Bioinformatics, Rhodes University PO Box 94, Makhanda 6140, South Africa; School of Biological and Marine Sciences, University of Plymouth, Drake Circus, Plymouth PL4 8AA, United Kingdom; School of Biomedical Sciences, University of Plymouth, Drake Circus, Plymouth PL4 8AA, United Kingdom; Department of Biochemistry, Microbiology and Bioinformatics, Rhodes University PO Box 94, Makhanda 6140, South Africa

**Keywords:** antimicrobial, sponge, biodiscovery, microbiome, deep sea

## Abstract

The deep sea is a largely unexplored extreme environment supporting a diverse biological community adapted to low temperatures and high pressures. Such environments support microbial life that may be a source of novel antibiotics and other drugs. Whilst this is often the case, many species with bioactive capabilities may be missed with traditional culturing methods. In this study, a total of 16 different concentrations and types of media were employed, to culture 389 bacterial isolates using Dilution to Extinction methods and Actinobacteria Directed Cultivation techniques. This generated 72 (18.6%) isolates with narrow and broad-spectrum activity against ESKAPE pathogens including *Escherichia coli* (*E. coli*), methicillin-resistant *Staphylococcus aureus*, and vancomycin-resistant *Enterococci*. We also report that an early-stage ‘One Strain Many Compounds’ approach can reveal a greater number of bioactive isolates that otherwise would have been missed; 12 isolates initially deemed ‘inactive’ were seen to exhibit activity towards *S. aureus* and/or *E. coli*. We emphasize the importance of a thorough initial screening method to capture bioactive isolates and show how selecting only morphologically distinct isolates for screening may result in species with promising bioactivity being overlooked. Our findings justify on-going investigation of *Pheronema* sponges for bioactive microbiota.

## Teaser

Bacteria that produce potentially new antibiotic compounds can be found in deep sea sponges like *Pheronema carpenteri* using a range of microbial growth conditions.

## Introduction

The need for new antimicrobial classes to be discovered cannot be understated; these vital drugs underpin our modern healthcare services. Traditionally, antimicrobials have predominantly been derived from natural products (NPs) from bacteria and fungi, mostly found within soil environments. However, the high rate of rediscovery and the significant costs associated with their development requires alternative environments and strategies to be used to reduce the risk of rediscovery (Devine et al. 2017). One solution to prevent re-discovery is to look for novel NPs in under-explored environments (Trenozhnikova and Azizan 2018, Srinivasan et al. 2021). Focusing on environments such as the deep sea reduces the rate of rediscovery because of unique adaptations required to survive in conditions such as low light, nutrient levels and temperatures, as well as high pressure and salinity—all factors that rarely need to be considered together for terrestrial samples (Saide et al. 2021).

Deep-sea sponge biodiscovery has gathered momentum in recent years, with their shallow water counterparts being well-researched and renowned for production of a plethora of bioactive NPs (Koch et al. 2021). These NPs have biomedical potential as anticancer, antimicrobial, anti-inflammatory and anti-viral leads (Varijakzhan et al. 2021, Hong et al. 2022, Khodzori et al. 2023). The success of biodiscovery programmes with shallow water sponges, coupled with the recent advancements in technology enabling discovery into deeper depths using remotely operated vehicles (ROVs), has motivated researchers to explore deep-sea sponges harbouring similar bioactivities. Demosponges are the most diverse phylum of sponge in the ocean and they are also one of the best researched, spanning from shallow water to the deep sea, from tropical oceans to the Arctic seabed (Steinert et al. 2020). Hexactinellida, or glass sponges, on the other hand, are much less well studied and are largely exclusive to the deep sea, except for locations such as the submarine caves in the Mediterranean or off the British Columbia coast where they form large reefs (Van Soest et al. 2012). These sponges are very fragile and sampling via trawling often results in significant damage to specimens and ecosystems, with specimens retrieved to the surface in pieces rather than as intact animals (Vieira et al. 2020). Regardless, studies into glass sponges have begun to increase within the last few years, with a particular focus on species that form aggregations, due to their functional role as a habitat and identification of these fields as Vulnerable Marine Ecosystems (Howell et al. 2016, Bell et al. 2018, Koch et al. 2021).

One common problem with culture-dependent studies is termed the ‘plate count anomaly’, suggesting that only 0.1%–1% of bacteria are cultivable under standard laboratory conditions, often with the same bacteria appearing frequently (Staley and Konopka 1985). This is thought to be due to classic incubation temperatures and media not being representative of the diverse environment the bacteria are sourced from, instead selecting for bacteria more able to adapt and grow in higher nutrient conditions (Mu et al. 2021). Similarly, many metabolites can be used for microbial inter-species communication and some bacteria are reliant on the production of essential metabolites from other species, living as a community instead of in isolation as they are grown commonly within a laboratory (Netzkar et al. 2015). Research has been conducted into varying incubation time and temperature, and nutrient type and concentration, with the view to replicate a marine environment and has led to successful cultivation of novel species (Rodrigues and Carvalho 2022, Lim et al. 2023).

Alongside altering the physical conditions of cultivation methods, the techniques themselves can also be changed. Moving away from standard plating techniques with nutrient-rich media to Dilution to Extinction (D: E) methods with oligotrophic media has been seen as a way of culturing novel and previously ‘unculturable’ marine bacteria (Benitez et al. 2021). Often D: E methods are also accompanied with diluted nutrients to replicate more closely the marine environment. In this method, roughly 1–10 cells or dilutions of 10^−8^–10^−10^ of neat suspensions, are inoculated into physically separated wells, usually a 96-well plate, to prevent or lower the chances of competition (Jung et al. 2021, Kim et al. 2021). As a result of D: E in low nutrient conditions, abundant but previously uncultured bacterial clades such as SAR11 (Rappé et al. 2002, Henson et al. 2018), SAR92 (Connon and Giovanni (2002), Cho and Giovannoni 2004), and others have now been isolated and grown in laboratories for genomic exploration. Jung et al. (2021) discovered that 48% of the bacterial species cultured through D: E methods were novel species, by employing oligotrophic conditions.

Often, initial screening efforts also result in the recovery of ‘low hanging fruits’, where one medium is employed, with one assay method, usually solid or liquid, then the isolates with activity are carried forward. Whilst this enables a rapid screening method to select for active isolates and a means of triaging, there is a bias towards the commonly isolated active isolates, often of species that have been previously investigated or are known to produce an antimicrobial that is already in preclinical development or even clinical use (Monciardini et al. 2014). Using the One Strain Many Compounds (OSMAC) approach during initial screening, although more time consuming, enables the identification of active isolates that would previously have been missed and results in a more extensive screen (Bode et al. 2002). Few studies have implemented OSMAC as a means of triaging; however, those that have used the approach early on in testing have seen promising results. Schwarz et al. (2021) discovered 590 novel mass features when implementing OSMAC early on in screening. Similarly, Williams et al. (2020) also employed low nutrients, carbon and pH conditions and triggered activity from 22 unique strains that had previously not exhibited inhibitory activity.

Here, we show how the culture conditions employed can directly affect the species cultured from a deep-sea sponge *Pheronema carpenteri* and demonstrate that an early-stage OSMAC can lead to a greater initial hit rate of isolates showing antimicrobial capabilities. Secondly, we show that selection of isolates based on morphological differences can also result in isolates of the same species that harbour a greater spectrum of activity being overlooked.

## Materials and Methods

### Sample processing and sponge taxonomy

**Figure 1.**
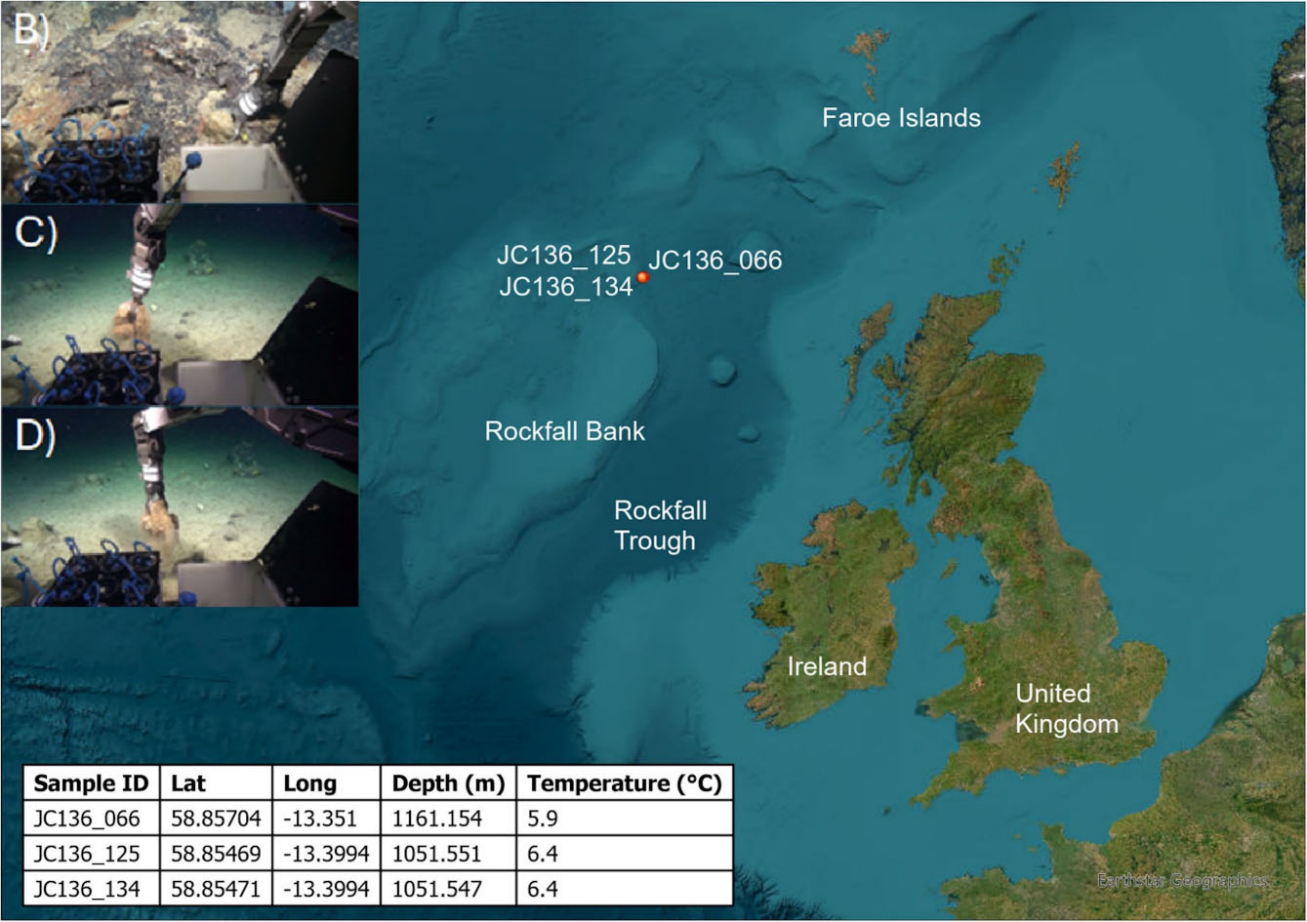
(A) Map showing the sampling sites of the JC136 sampling events and the location of each sponge, collected from the North Rockall Bank, namely the George Bligh Bank with the bottom left table showing the sample coordinates, temperature and depth. (B)–(D) show *in-situ* sampling events of each sponge: (B) JC136_066, (C) JC136_134, and (D) JC136_125.

Sponge samples were collected from the Northeast Atlantic Deep Sea as part of an NERC-funded DeepLinks project in 2016 (RRS James Cook—JC136; Fig. 1). During these cruises, a range of samples were collected, including many species of deep-sea sponges (below 200 m). Sponges were photographed in-situ and collected using ROVs. When the ROV surfaced, sponge samples were placed into a bucket containing surface seawater and processed on the ship. Sponge samples were photographed, and a small tissue sample was taken for separate genetic analysis. Remaining tissue was then placed into a zip-lock bag, frozen at −20°C for the duration of the cruise, transported to the University of Plymouth on dry ice and frozen at −80°C. Internal and external morphological observations (body shape, size, spicules) were undertaken to assign them as *Pheronema carpenteri* sponges, as outlined by Hesketh-Best et al. (2019).

### Sponge sample processing and bacterial isolation

Sponge tissue was cut into 1 cm^3^ cubes under aseptic conditions and washed three times with sterile Instant Ocean (IO) (instantocean.com), to remove any debris and loosely attached bacteria. The chosen cut site was located within the mesohyl to reduce the risk of contamination from sea water or sediment. The sponge sample was submerged in 9 mL of autoclaved sterile water and homogenized using a sterile mortar and pestle. The homogenate was then serially diluted 10^−1^–10^−8^ in each medium condition (see below; MA, MA 1:2, R2A, R2A 1:10, NB, NB 1:100) for D: E experiments and standard direct plate comparisons. For Actinobacteria Directed Cultivation (ADC; see below), the same steps were taken as described above but the sponge homogenate was diluted in IO instead of a particular broth medium.

### Nutrient ‘Rich’ (eutrophic) and nutrient ‘Poor’ (oligotrophic) media preparation

For D: E cultivation experiments, the following media were used: (i) Marine Broth 2216 (Merck) undiluted(MB) and diluted 1:2 (MB 1:2) (50% of manufacturer’s suggested concentration); (ii) Reasoner’s 2A (R2B) liquid medium (Merck) undiluted and diluted 1:10 (10% of manufacturer’s suggested concentration); and (iii) Nutrient Broth (NB) (Merck) undiluted and diluted 1:100 (1% of manufacturers suggested concentration). All dilutions were carried out with deionized water. For solid agar comparisons, the same nutrient conditions stated above were employed with the addition of agar (15 g/L) (Merck). Media were sterilized by autoclaving at 121°C and 15 psi for at least 15 minutes. D: E cultivation was carried out according to Jung et al. (2021) in 96-well plates with a total volume of 200 μL and with the addition of cycloheximide (25 μg/mL). Plates were incubated at normal atmosphere for 8 weeks at 4°C and 28°C without shaking and the percentage coverage of the bottom of each well was visually estimated, an average of all wells within each nutrient condition was then taken, weekly from Week 5. At Week 8, the final time point, 10 μL of the content of each well was used to inoculate a corresponding agar plate [e.g. NB inoculated onto Nutrient Agar (NA), etc.] and plates were left to incubate to allow for the selection of isolates and diversity assessment (D: E step 2). This was done for both 4°C and 28°C plates, incubated at the same corresponding temperatures, with 4°C and 28°C samples incubated for 8 weeks, both with weekly checks. Isolates were selected based on the level of abundance on the plate, and therefore, multiple colonies with the same morphology were selected if that was representative of the plate and they were streaked until axenic. This method was employed to assess whether selection based upon morphological differences could miss bioactive isolates (D: E step 3). To determine the effect of nutrient conditions on the rate of growth, an average of all serial dilutions in the D: E experiment was taken, and a single value was used to represent each condition and each sponge.

### OSMAC media preparation and soft agar overlay

Undiluted and diluted media were created as described stated above. For OSMAC studies, pH was adjusted to either pH 9 or pH 5 with sodium hydroxide (NaOH, VWR) or hydrogen chloride (HCl; Fisher Scientific, UK), respectively, before autoclaving. Carbon and nitrogen sources were altered using M9 minimal medium (Supplementary Table 1) as a base, supplemented with one of the following at 10 g/L: casein (Fisher Scientific, UK); meat extract (Fisher Scientific, UK); yeast extract (Fisher Scientific, UK); glucose (Merck, UK); or sucrose (Merck, UK). A control of M9 medium without additional nitrogen or carbon was assessed to see whether basal M9 medium could stimulate bioactivity. Single isolates were stabbed into each medium (as stated above) using autoclaved wooden cocktail sticks and incubated at 28°C for 5 days before soft-agar overlays were applied containing *Staphylococcus aureus* 48 491 or *Escherichia coli* NCTC 10418. Pathogens were streaked from −80°C cryopreserved glycerol stocks, onto a fresh Mueller Hinton Agar (MHA) plate and incubated overnight at 37°C, when single colonies with the same morphology were inoculated into Mueller Hinton Broth (MHB) and grown overnight at 37°C, with shaking at 120 rpm. These overnight broths were adjusted to 0.05 OD_600_/mL and were used to seed 0.75% soft agar that was cooled to below 50°C and 20 mL was poured onto the OSMAC plates. All plates were left to rest at room temperature for 30 minutes before being placed in an incubator overnight at 37°C (D: E step 6a).

### ADC using solid agar

Actinobacteria are responsible for producing the majority of antibiotics available on the market today and so were specifically targeted using a range of media in this solid agar plate cultivation approach (De Simesis and Serra 2021). Media abbreviations in the text are as follows: SCA, Starch Casein Agar; ISP2, International Streptomyces Project 2 agar; OM, Oatmeal Agar; ACT, Actinomycete Agar; HVA, Humic Vitamin Agar. IO was added (3.3% w/v) to some of the above. A full list of all media conditions can be found in the Supplementary Material (Supplementary Table 1). All media used were supplemented with the addition of antifungal agents nystatin (25μg/mL) (Fisher Scientific, UK) and cycloheximide (Fisher Scientific, UK) (25 μg/mL) and the antibacterial nalidixic acid (10 μg/mL) (Fisher Scientific, UK), which were filter-sterilized and added to cooled media following autoclaving. The homogenate and IO as stated above, was spread onto each agar plate (100 μL) and left to incubate aerobically for a total of 12 weeks at 4°C, 15°C, or 20°C and checked for new isolates every 2 weeks, after Week 4 (ADC step 1). Colonies of distinct morphologies were selected for axenic culture on ISP2 agar. If selected isolates failed to grow on ISP2 agar, they were cultivated on the solid medium of their original isolation and subsequently sub-cultured until axenic (ADC step 2).

### Antimicrobial bioactivity screening

Several methods were used to assess the antimicrobial bioactivity of isolates generated using the above culture-based methods. Cell suspensions of pathogenic indicator organisms from the ESKAPE group were used in screens. These were prepared from an overnight culture and diluted to an OD_600 nm_ of 0.05. Indicator strains were from the following list: *Acinetobacter baumannii* ATCC 19606*, Enterococcus faecalis* NCTC 12697*, E. coli* NCTC 1048*, Klebsiella pneumoniae subsp. pneumoniae* NCTC 9633*, Micrococcus luteus* NCTC 2665*, S. aureus* 48491 (in house strain)*, Pseudomonas aeruginosa* PA01, and *Enterococcus faecalis* ATCC 19433 (vancomycin-resistant). *Candida albicans* 379 (in-house isolate) was used as a representative yeast and grown in MHB medium for 48 hours. ESKAPE pathogens were used as they are shown to be the leading cause of nosocomial infections worldwide (De Oliveira et al. 2020). Initially, all sponge associated isolates were screened in triplicate against *M. luteus, S. aureus*, and *E. coli*. If activity was observed in two or more repeats, then the full spectrum of the above indicators was tested against for bioactivity (*n* = 3).

Simultaneous antagonism assays were used for the initial screening process towards the three primary screening pathogens (*M. luteus, S. aureus*, and *E. coli*) using the methods outlined in Koch et al. (2021) (D: E step 5).

### Well diffusion assay

This method was used to assess production of bioactive compounds in broth media. Isolates derived from the ADC methods, were inoculated into 10 mL of ISP2 broth, and left to incubate for 14 days, with shaking at 120 rpm and 20°C. Overnight cultures of the pathogens were grown at 37°C and 160 rpm and then adjusted to an OD at 600 nm of 0.05 and spread plated onto MHA square plates using a sterile glass spreader to create a confluent lawn and left to dry. A volume of 1 mL was taken from the ADC isolate broths after 14 days and centrifuged at 12 000 × *g* for 10 minutes before 6-mm diameter holes were made in the indicator lawn plates using a sterile borer. The centrifuged cell-free supernatant (50 μL) was then pipetted into the wells. Plates were left at room temperature for 30 minutes before being placed at 37°C to incubate overnight (ADC step 3).

### Soft agar overlay

To ensure all isolates had been assayed for bioactivity, all culture plates from D: E and ADC cultivation studies, were tested for activity with a soft agar overlay, using *M. luteus* at OD_600 nm_ of 0.1, to ensure all colonies had been tested for activity before discarding plates.

### Active isolate prioritization and triaging

Isolates in D: E methods were not selected based upon being morphologically distinct; therefore, there was the risk that many of the isolates could be the same species. However, as this study was more focussed upon discovering bioactivity, isolates that looked morphologically similar were grouped and assayed for bioactivity. Isolates from that group with the greatest bioactivity (largest zone sizes, or activity towards Gram-negative pathogens), were then carried forward for further study (D: E step 6).

In ADC, isolates exhibiting the most promising level of activity were prioritized for screening as the initial selection of isolates from culture had been based upon morphology. All selected isolates were then sent for 16S rRNA gene sequencing (see below) to assist in identification.

### OSMAC screening of triaged isolates

To increase the number of active isolates, the OSMAC approach was undertaken. Culturing conditions were altered in a variety of ways to create a bacterial stress response, with the intention of triggering the expression of silent biosynthetic gene clusters (BGCs) and production of bioactive secondary metabolites. Thirty morphologically distinct isolates, having shown no previous activity from D: E conditions, were selected for OSMAC studies (D: E step 6a). The previous nutrient conditions (MA, MA 1:2, R2A, R2A 1:10, NA, NA 1:100) were used at pH 5 and 9 to determine whether nutrient conditions coupled with pH could trigger the secretion of active compounds. Similarly, altering carbon and nitrogen at a pH 7 was used to assess whether different sources of these elements triggered expression of bioactivity (D: E step 7a). All plates were incubated at 28°C for 5 days before soft agar overlays with *S. aureus* and *E. coli* (D: E step 8a).

### DNA extraction and 16S rRNA gene amplification of prioritized strains

Prioritized strains were sent for 16S rRNA gene sequencing (LGC Genomics Ltd, Germany). A single colony of each isolate, including *E. coli* as a positive control, was placed into 100 μL of deionized water, frozen at −80°C for 30 minutes, and then heated to 60°C for 30 minutes. PCR reactions were set up consisting of a Master Mix containing 2 μL from the above crude lysis method added to 1X Dream Taq (Thermo Scientific, UK), 0.4 μM forward primer 27F (5’-AGAGTTTGATCATGGCTCAG-3’) and reverse primer 1492R (5’-GGTTACCTTGTTACGACTT-3’) (Eurofins, Germany) (Weisburg et al. 1991), with ddH_2_0 up to a total volume of 50 μL. The following thermal cycler (Applied BioSystems, UK) program was used: initial denaturation (1 cycle) at 95°C for 10 min; amplification (35 cycles)–30 seconds at 95°C, 7 seconds at 53°C, and 1 minute at 72°C; final extension (1 cycle) 7 minutes at 72°C. Visualisation of amplified DNA was conducted using gel electrophoresis (1% w/v agarose gel in 1X Tris-acetate-EDTA (TAE) buffer) at 100 V for 45 minutes. PCR products were cleaned up at LGC Genomics Ltd Germany and sequence data was received in *.scf format for assembly using Geneious Prime 2024 version 10.2.3 (https://www.geneious.com).

### Phylogenetic tree building and bacterial sequence classification

Forward and Reverse strands of the 16S rRNA gene were sequenced, trimmed (to 1400–1500 bp) and aligned *de novo*, producing a consensus sequence in Geneious Prime. Sequences were compared to the NCBI BLAST Nucleotide collection database for species identification and similarity. A multifasta file was then created and a multiple sequence alignment (MSA) generated using Clustal Omega with default settings (https://www.ebi.ac.uk/jdispatcher/msa/clustalo) and the tree was visualized and edited in iTol (version 7) (https://itol.embl.de).

### Statistical analysis

Statistical analysis with a Chi-squared test was carried out using Prism 10.4.0 (GraphPad Software, Boston, USA www.graphpad.com). Chi-squared tests involved comparison of data points from 4°C with 28°C to determine whether the results were statistically significant (*P* < 0.05). The same Chi-squared test was then carried out comparing all oligotrophic results to eutrophic results to determine significance (*P* < 0.05).

## Results

### Oligotrophic and eutrophic conditions affect the level of growth observed

**Figure 2.**
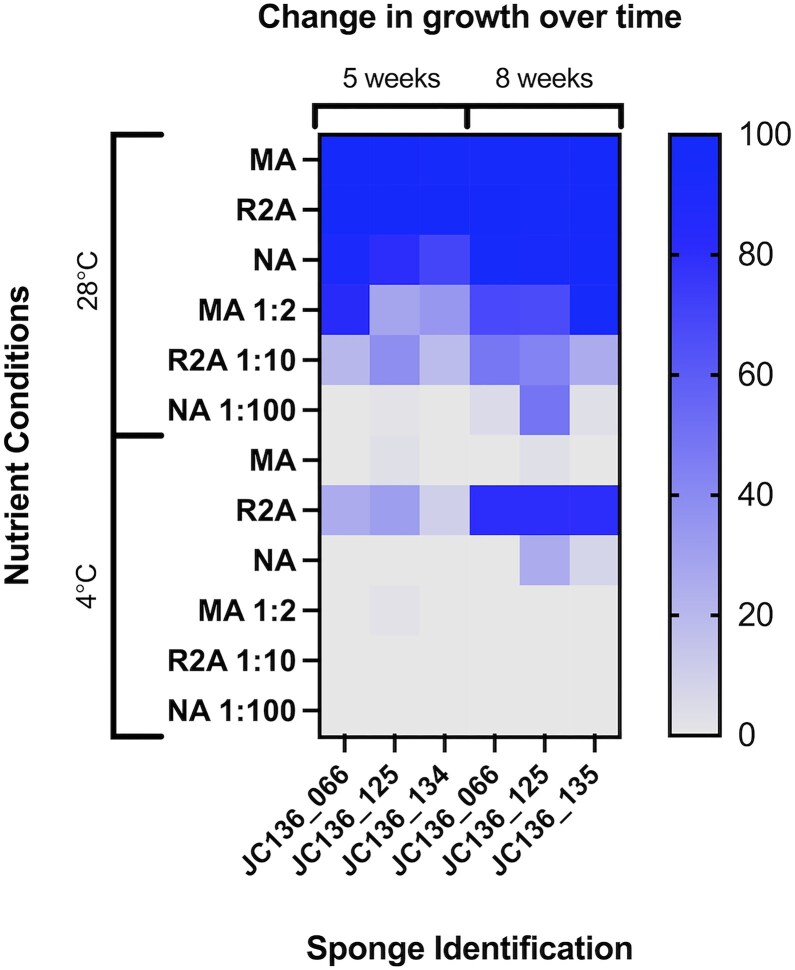
Change in growth over time across nutrients and temperature. Heat map showing the effects of temperature, nutrient levels, and incubation duration on the amount of growth of *Pheronema carpenteri* sponge-derived bacteria. Colours in chart and heatmap represent the average level of growth (% coverage of surface of assay wells) in triplicate wells.

Across both temperatures used in the D: E experiment, nutrient availability directly correlated with the extent of growth observed within the well. Overall, there was a trend at 28°C, suggesting that nutrient concentrations directly affected the level of growth, with undiluted medium reaching upwards of 80% bacterial coverage on the bottom of the well by Week 5, except for Sponge JC136_134 (70%—NA; Fig. 2). The rate of growth appeared to be slowed by oligotrophic conditions where the level of growth ranged from 30% to 70% in MB 1:2, to 0%–5% in NB 1:100. To an extent, this trend was also shown at 4°C where eutrophic wells had signs of slight growth in MB and NB (0% and 10%–30%, respectively) by Week 5. Then R2B had the most growth of 10%–80% in the same time period. Oligotrophic conditions at 4°C had no growth observed across the whole experiment.

At temperatures of 28°C, most wells had reached over 80% by the first time point (five weeks) and 100% by 8 weeks, suggesting maximal growth conditions for the dominant species. Time also enabled growth to be evidenced in oligotrophic conditions where there was an increase in growth with MB 1:2 from 30% to 80% growth at Week 5, to 70%–100% by Week 8. This trend was also observed for R2B 1:10 20%–40% at Week 5, to 30%–40% by Week 8, suggesting that limiting nutrients at this temperature does require extended incubation periods for full growth to be achieved, and using longer incubation could have been beneficial. At 4°C, the length of incubation did not appear to influence the growth in any wells, except with R2B media where there was growth of 10%–40% at Week 5 and 80% at Week 8. Similarly, wells with NB had no growth at Week 5 but by Week 8, some wells had signs of growth (10%–30%), suggesting that low temperatures and limited nutrients require incubation periods of more than 8 weeks (Fig. 2). A chi-square test revealed eutrophic and oligotrophic conditions at 28°C had a significant difference (*P* ≤ 0.0001) in the amount of growth; however, eutrophic and oligotrophic conditions at 4°C did not have a significant effect on growth (*P* ≤ 0.05).

### Temperature affects the level of observed diversity on solid agar

**Figure 3.**
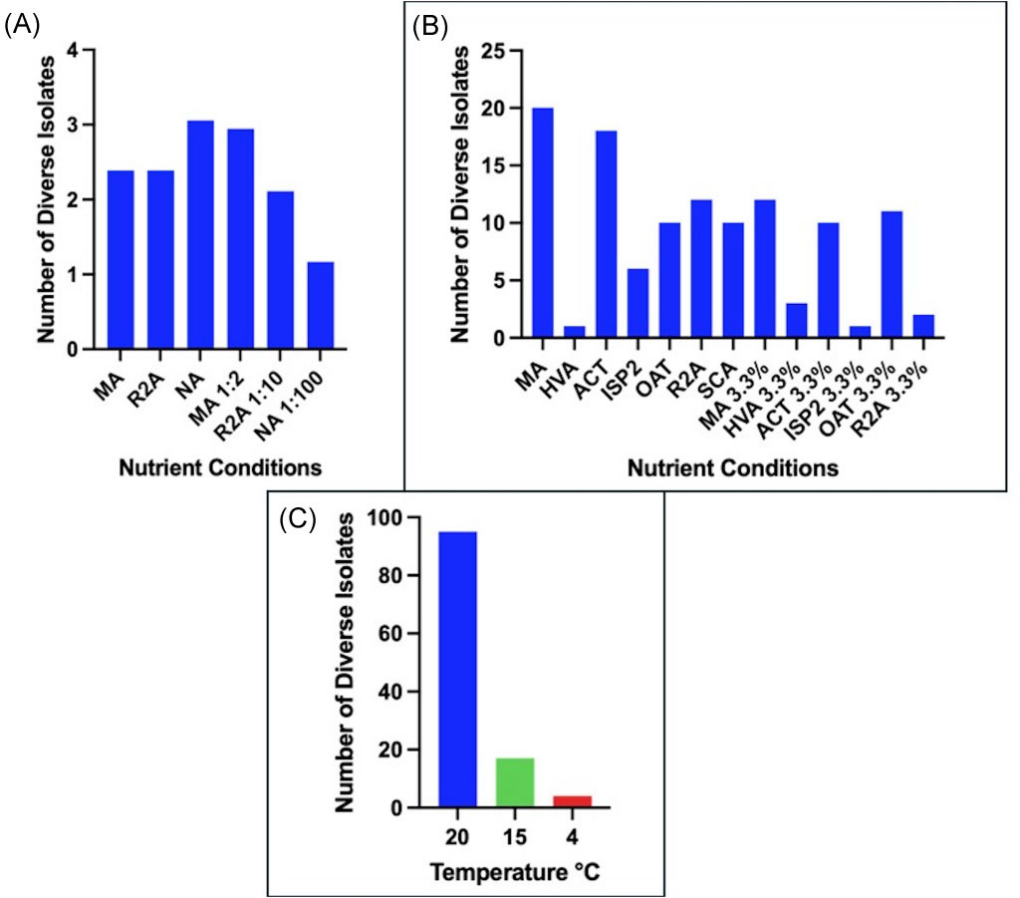
(A) Average diversity observed across dilution to extinction agar plates after incubation. (B) The number of morphologically distinct isolates from ADC strategies employing nutrient conditions with and without IO at 3.3% w/v. (C) The effect of temperature on the level of diversity in ADC. Counts represent the average across three replicate experiments. Boxes highlight ADC conditions.

Not surprisingly, the lower temperature (4°C) appeared to substantially reduce the growth rate and diversity of bacterial isolates in both D: E and ADC conditions, with only a small number of isolates growing within the study time frame. Conversely, higher temperatures showed an abundance of bacteria, although some plates were dominated by one species; this was evident especially in D: E (Fig. 3A, B). Decreasing temperature in ADC conditions correlated with a decrease in isolate numbers, with the greatest level of diversity shown at 20°C (95 isolates) (Fig. 3C). The media conditions also correlated with the species of bacteria isolated; generic media such as R2A or NA grew colonies morphologically resembling *Bacillus* species, whereas SCA or ISP2 characteristically harboured slower growing, chalky Actinobacteria isolates. The difference in temperature had a significant impact (*P* ≤ 0.001) on growth when all conditions were compared using a chi-squared test.

The greatest level of diversity was shown in MA and ACT with 20 and 18 distinct colony morphologies recovered, respectively. The lowest level of diversity was evidenced with just a single colony recovered on both ISP2 3.3% and HVA agar. The presence of 3.3% IO seemed to decrease the diversity present on the plate in all cases except for OAT and HVA where there was a slight increase in diversity (Fig. 3B). Diversity was low throughout all D: E conditions, with the most diversity seen with NA and MA 1:2 averaging three distinct morphologies per plate at 28°C (Fig. 3A).

### Bacteria recovered from dilution to extinction and ADC methods and their bioactivity

Comparison between D: E and ADC techniques cannot be made due to the nature of isolate selection and bioassay testing. D: E isolates were chosen based on the relative abundance observed on the isolation plate, not considering whether isolates were morphologically distinct whereas ADC isolates were selected if morphologically distinct, so no conclusion as to the best method for active isolates can be drawn.

**Figure 4.**
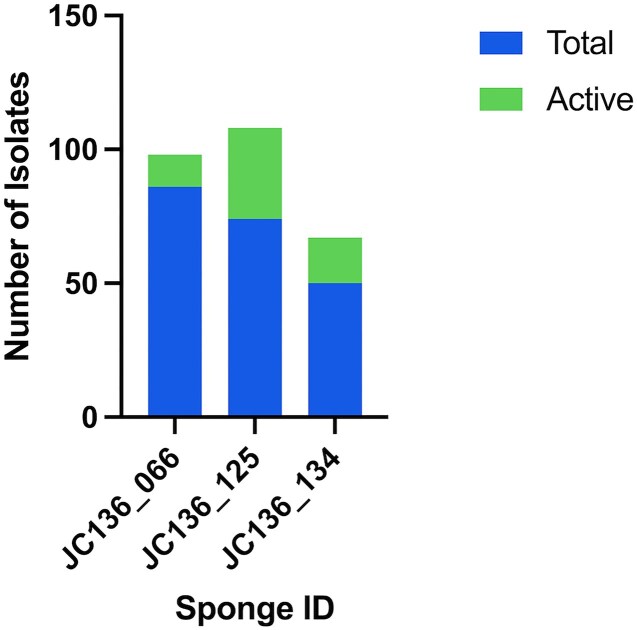
Stacked bar chart of sponges JC136_066, JC136_125, and C136_134 with the number of selected isolates and the portion of those active from D: E conditions.

In total, 273 marine sponge isolates were recovered from three deep sea *P. carpenteri* sponges, under various D: E conditions. Of these, most isolates were from sponge JC136_125 followed by JC136_066 and finally sponge JC136_134, with 108, 98, and 67 isolates recovered from each, respectively (Fig. 4). Of those isolates, 31% were active from sponge JC136_125, 12% from JC136_066, and 28% from sponge JC136_134. A further 116 morphologically distinct isolates were cultured using ADC agar with active isolates generally less frequent with 9% (10) of isolates active in total across the three sponges.

### Impact of nutrient conditions on active isolate recovery

**Figure 5.**
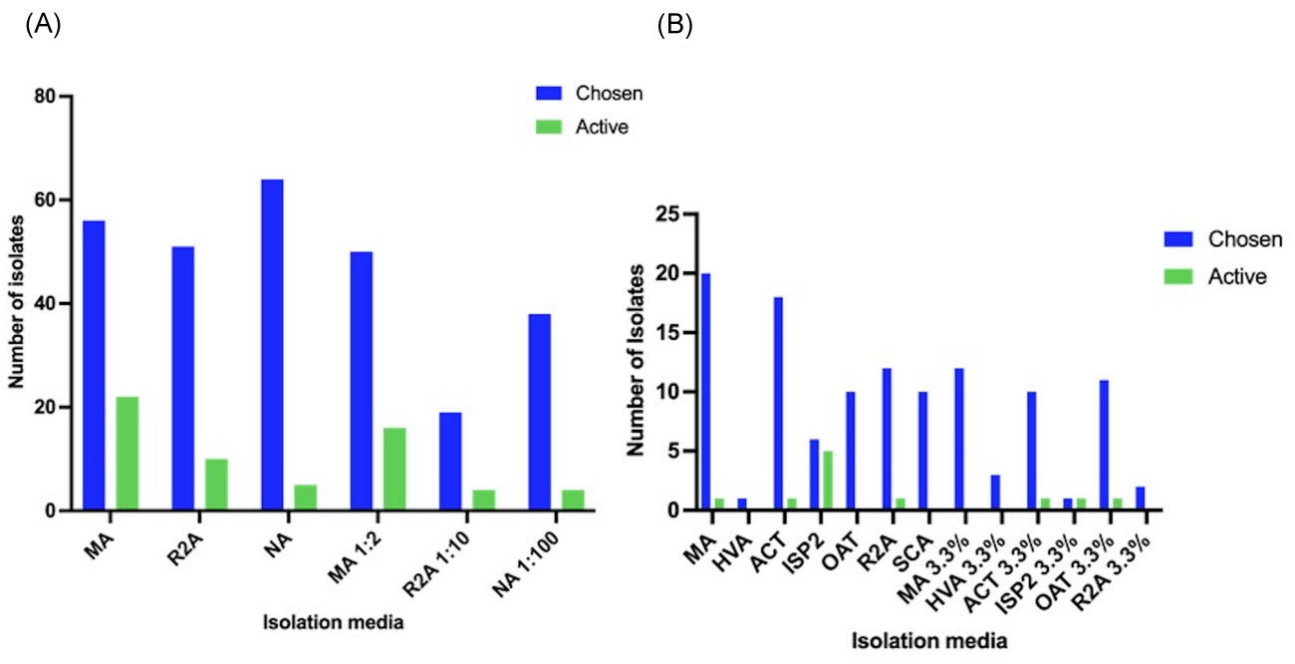
(A) Number of isolates chosen over six nutrient conditions used for the recovery of sponge-associated bacteria and an indication of those that were active in two or more repeats of antimicrobial screening assays. These isolates were recovered during dilution to extinction experiments. (B) The number of morphologically distinct bacteria selected over 13 nutrient conditions and the distribution of activity across nutrients in ADC. (A) and (B) isolates are designated as 'Chosen' or 'Active' as indicated in the key.

After incubation on solid agar and subsequent bioactivity testing, Nutrient agar (NA), although having yielded the highest number of isolates, had the lowest number of active isolates present, both in eutrophic and oligotrophic D: E conditions (five and four active, respectively). The top 3 nutrient conditions for recovery of active isolates were MA, MA 1:2, and R2A with 22, 17, and 9 hits, respectively (Fig. 5A). In ADC conditions, MA and Actinomycete Isolation agar (ACT) had the highest level of diversity but there was only one isolate active from each medium. The nutrient condition with the highest level of activity was ISP2, with five out of six isolates exhibiting bioactivity from morphologically distinct isolates.

### Bioactivity was observed towards Gram-negative and Gram-positive pathogens in dilution to extinction conditions

**Figure 6.**
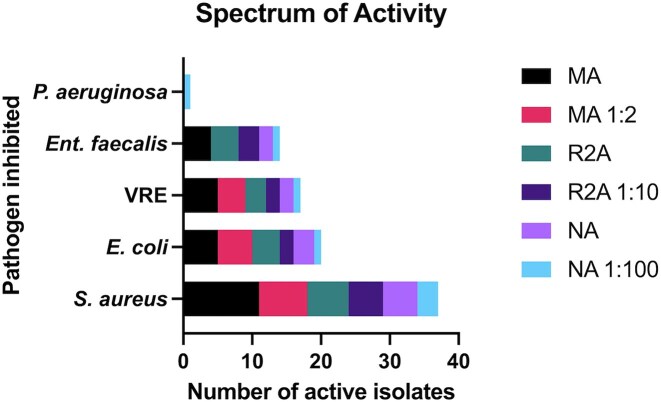
Results of simultaneous antagonism assays across a panel of ESKAPE pathogens to explore the spectrum of activity of 72 isolates from dilution to extinction experiments and to guide isolate prioritization. VRE; vancomycin-resistant *Enterococcus*.

A total of 72 isolates had activity in *n* = 2 or 3 assays towards *S. aureus* or *E. coli* were carried forward for further testing against ESKAPE pathogens and a representative yeast. No isolates showed activity towards *A. baumanii, K. pneumoniae*, or *C. albicans*. A total of 39 (54.1%) isolates showed activity, predominantly towards *S. aureus*. Of those active isolates, 14 (35%) inhibited the growth of two or more pathogens, and one inhibited three pathogens: Vancomycin-resistant *Enterococcus faecalis* (VRE)*, Enterococcus faecalis*, and *Pseudomonas aeruginosa* (Fig. 6).

### Cultivation techniques directly influence the species diversity

**Figure 7.**
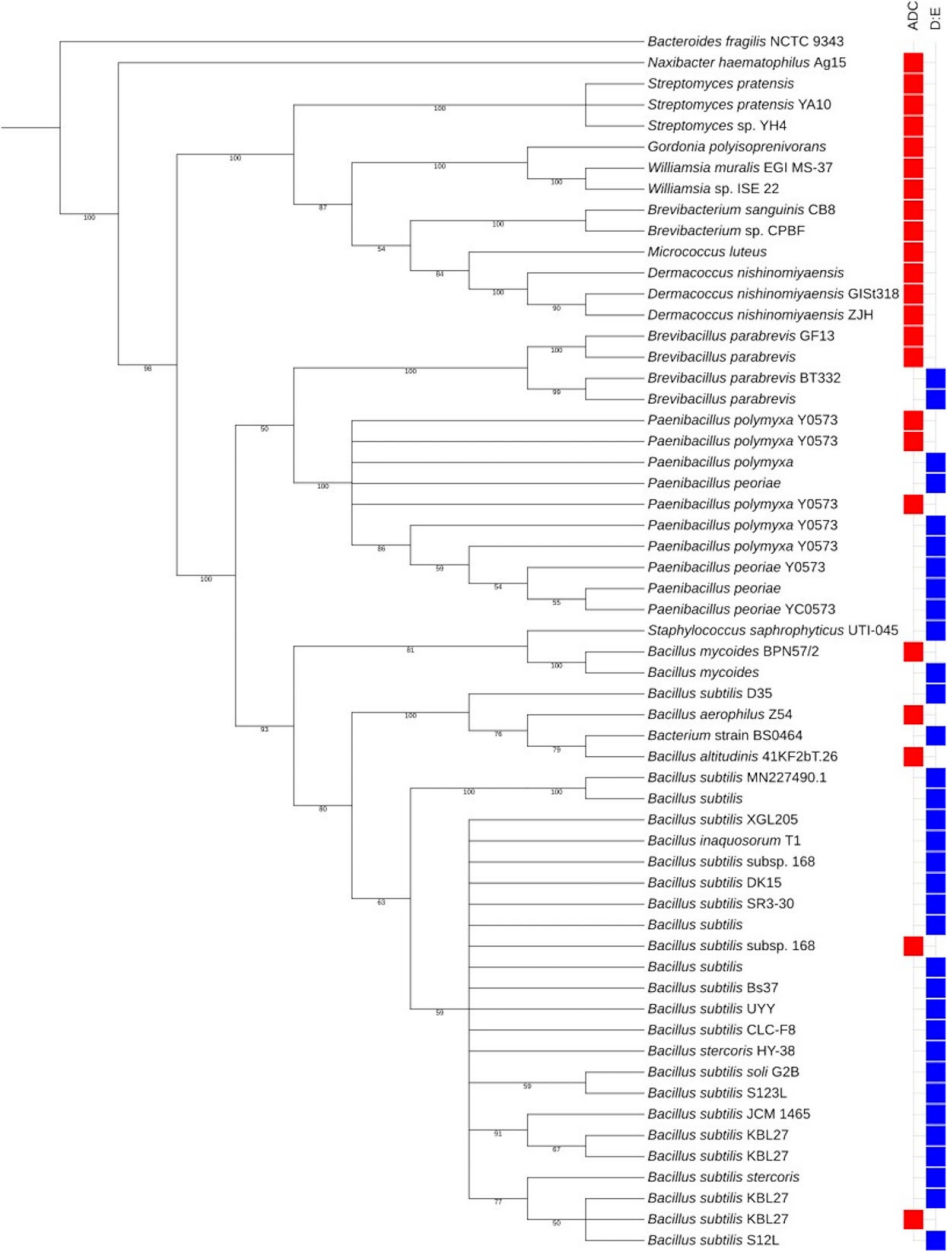
Phylogenetic tree presenting the best database hits for 16S rRNA gene sequences of active isolates cultured using ACT-directed cultivation (ADC) and dilution to extinction (D:E) methods.

The selection of isolates sent for 16S rRNA gene sequencing was based upon both morphology and bioactive capability and, therefore, should not be considered entirely representative of the community investigated. A total of 56 isolates were chosen for 16S rRNA gene sequencing and the findings are presented in a phylogenetic tree (Fig. 7). There was a clear correlation between the species recovered and the culturing technique employed as isolates most closely related to strains of the genus *Bacillus* were dominant with the D: E method (Fig 7: blue squares). With the exception of a few strains identified as related to ‘*Bacillus* species’, *Bacillus* isolates were identified as *Bacillus subtilis* in D: E conditions, showing dominance of one species in particular. Isolates of members of the genus *Actinobacteria* were dominant with ADC-directed cultivation (Fig. 7: red squares), which suggests that the method and nutrients used directly affect the species cultured. Although *Actinobacteria* were the dominant genus, there were still *Bacillus* species cultivated in ADC directed cultivation and vice versa, indicating the capability of some species to grow across multiple isolation methods. A full list of the species isolated can be found in Supplementary Table 2.

### Isolates of the same species can exhibit varying levels of activity

In some cases, the 16S rRNA gene sequence of some active isolates were most similar to the same database reference strain. For example, isolates 152, 278, and 316 were most similar to *Bacillus subtilis* KBL27 (Table 1). Similarly, isolates 94 and L15 were most similar to *B. subtilis subsp*. 168. In both examples, although isolates were very similar at the level of 16S rRNA gene sequencing, the level of activity was different; strain 316 showed activity towards *S. aureus* only, whereas strain 152 had activity towards *S. aureus, E. coli*, and VRE. Similarly, strain L15 exhibited *S. aureus* activity, whereas strain 94 inhibited *S. aureus, E. coli, E. faecalis*, and VRE (Table 1).

**Table 1.** Species level identification, based on full-length 16S rRNA gene sequencing, of isolates having different antimicrobial capabilities but similar colonial morphology.

Isolate number	Culture media used for isolation	Activity	Morphology	Best species match based on 16S rRNA gene sequence	Bp match to best hit sequence	BLAST % match
152	NA	*Staphylococcus aureus, E. coli*, VRE	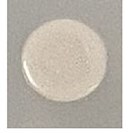	*Bacillus subtilis* KBL27	1437	100
278	MA	*Staphylococcus aureus, E. coli*	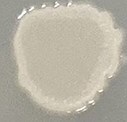	*Bacillus subtilis* KBL27	1437	100
316	R2A	*Staphylococcus aureus*	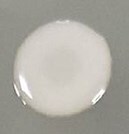	*Bacillus subtilis* KBL27	1439	99
94	R2A 1:10	*Staphylococcus aureus, E. coli, E. faecalis*, VRE	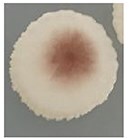	*Bacillus subtilis subsp*. 168	1437	99
L15	MA	*Staphylococcus aureus*	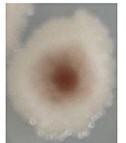	*Bacillus subtilis subsp*. 168	1430	100

### An OSMAC approach triggers antimicrobial production

Many bacteria are known to harbour ‘cryptic’ BGCs, regions capable of producing secondary metabolites in times of stress. An OSMAC experiment was undertaken through the manipulation of nutrient conditions, pH, carbon and nitrogen sources and the addition of chemical elicitors with the aim of activating cryptic loci. Unfortunately, the use of meat and yeast extract supplementation returned inconclusive results with extensive overgrowth of dominant species by Day 5 (*n =* 3; data not shown).

The remaining six isolation media under eutrophic and oligotrophic conditions were employed at pH 5 and pH 9, to determine whether a change in nutrients and acid/alkaline pH could stimulate the production of antimicrobial compounds. Screening revealed that activity was triggered on seven occasions at pH 5 with three isolates specifically only active at the lower pH. Similarly, activity was observed on eight occasions at pH 9 with two isolates specific to that pH (Table 2). At pH 5, the nutrient condition responsible for triggering most activity was MA 1:2 (three active), followed by MA and NA both with two active isolates (Table 2). MA and NA were the most optimal conditions at pH 9 with three hits, followed by NA 1:100 with two. When analysing both pH and nutrients combined, it appears all but two isolates have both pH and nutrient specificity, only exhibiting activity with one nutrient condition at either a pH 5 or pH 9. One isolate showed activity in two nutrient conditions at pH 5 and one isolate showed activity in three conditions at pH 9, leaving four isolates with both pH and nutrient-specific requirements for activity to be observed. Isolate 319 was the most broadly productive, being active in four conditions. Interestingly, no isolates showed activity on R2A undiluted or at the 1:10 dilution, regardless of pH. Only three isolates displayed activity towards *E. coli*, and all were stimulated in pH 9 MA conditions. Overall, there were 10 hits in nutrient-rich conditions, with both pH combined, and 5 active in oligotrophic conditions.

**Table 2.** Nutrient conditions and pH triggering bioactivity in *Pheronema* sponge-associated bacteria*

	pH 5						pH 7				pH 9					
Isolate number	MA	R2A	NA	MA 1:2	R2A 1:10	NA 1:100	Control	Casein (N)	Sucrose (C)	Glucose (C)	MA	R2A	NA	MA 1:2	R2A 1:10	NA 1:100
32			S													
33			S					S								
134									S							
165								S								
175				S												S
182	S			S							S/E					
193				S												
197										S						
202										S						
223											S/E		S			
230													S			
319	S										S/E		S			S
Total at each media	2		2	3												
Total at each pH	7						5				8					

*Staphylococcus aureus (S) or *E. coli* (E) with activity against both *S. aureus* and *E. coli* noted as S/E at pH 5, 7, and 9. (C) and (N) indicates whether the additive is of carbon or nitrogen source. The numbers 1:2, 1:0, or 1:100 show the dilution factors of the media used (MA, R2A, or NA).

At pH 7, five isolates exhibited activity towards *S. aureus*; use of both casein and glucose yielded two active isolates and one was recovered from sucrose supplemented media. The control medium, M9 Minimal Media, with no added carbon or nitrogen sources, had no impact on activity. Three isolates (134, 197, 202) were carbon source-specific, and two (33, 165) were nitrogen specific, with no other activity observed in varied pH, eutrophic, or oligotrophic conditions. No activity was observed towards *E. coli* alone and no condition stimulated activity in all strains.

## Discussion

There is growing interest in deep sea sponges and other marine niches as a source of novel chemical matter with bioactive potential. In this work, we sought to investigate the impact of growth conditions on the diversity of cultivable sponge-associated bacteria exhibiting antimicrobial activity, with the aim of improving the recovery of novel species and reducing the likelihood of rediscovery of known compounds. Our focus has been on the *P. carpenteri* glass sponge, as this has not been studied extensively previously.

Koch et al. (2021) showed that D: E methods applied to *P. carpenteri* samples produced a number of diverse isolates, leading us to employ D: E methods in this study. Within our study the focus was upon incorporating oligotrophic (diluted) conditions to explore whether this could encourage the growth of diverse species, whereas Koch et al. (2021) explored the use of various medias supplemented with and without carnitine and the effects of pressure. Our study also used a temperature of 28°C to target *Actinobacteria *, a phyla known for wide bioactivity capabilities, which have optimal growth rates at temperatures of 25°C–30°C. D: E conditions at this temperature created optimal conditions for fast growing dominant species, with cell growth within the 96-well plate exceeding 50% when elevated temperatures and nutrient rich conditions were coupled together. This outcome had been expected and has been seen in previous studies; therefore, serial dilutions of the inoculum were made to reduce the microbial load, reduce competition, encourage diversity, and allow recovery of slower growing species—though it appears that these efforts were unsuccessful (Hames-Kocabas and Uzel 2012). The overgrowth of dominant species could have been due to the ‘competition phenomena’ where the level of competition is too great for any increase in diversity to be observed and so one species grows the fastest and becomes dominant (Benitez et al. 2021). Sequencing of the 16S rRNA gene, revealed that *Bacillus* species were the most dominant when using D: E techniques. This dominance has been shown in previous data, in a review by Dat et al. (2021) it was stated that generally the most commonly cultured genera from sponge species were *Pseudovibrio* (14.3%) followed by *Bacillus* (10.2%) and *Streptomyces* (9.1%). One potential explanation for the lack of *Pseudovibrio* in our study could be the choice of media selected; although *Pseudovibrio* species thrive in oligotrophic conditions, they may have been outcompeted by the dominant species, e.g. *Actinobacteria* or *Bacillus*. Examination of 16S rRNA amplicon data from our group with the same sponge samples (*Unpublished data*) showed *Bacillus* to be non-dominant in these sponges, leading to the assumption that this species behaves ‘opportunistically’ with the chosen cultivation nutrients selecting for these bacteria. Similarly, Rodrigues and Carvalho (2022) suggested that *Bacillota* make up 0.06% in culture-independent studies, compared to 20.6% in culture-dependent studies of marine samples. *Bacillus* species can grow quickly in nutrient-rich media and outcompete slow-growing species, therefore, the selection of media in addition to the culturing method used appears to have impacted the species of bacteria cultured within this study. It is also possible that *Bacillus* species spores will have survived sample recovery and storage processes more robustly that vegetative cells of other genera. Similarly to our observations, previously studies have reported *Bacillus* species to harbour antimicrobial capabilities. Activity towards Gram-negative species has been observed from *B. subtilis* such as polymyxin, difficidin, and bacitracin previously (*Santhi et al. 2017*). Similarly, bioactive *Bacillus* sp. have been recovered from the sponge *Dysidea avara* (Li et al. 2007). *Bacillus* sp. isolated from sponge class Homoscleromorpha showed evidence of having activity towards multidrug-resistant strains and these could be an additional source of bioactive compounds (Freitas-Silva et al. 2020). The presence of a large number of bioactive *Bacillus* isolates in the current study is promising and warrants further investigation of Demosponges and members of other sponge classes.

Low temperatures resulted in a significant decrease in growth across all nutrient conditions except for R2A. Low temperatures and oligotrophic conditions decreased the rate of growth significantly, with very little growth being observed within the 8-week incubation period. Research suggests low temperatures and low nutrients do usually enable the cultivation of isolates, but in this study, very low numbers were generated. This could be attributed to the incubation period being too short for bacteria to grow under these conditions. Connon and Giovanni (2002)and Kong et al. (2022) were able to see growth in up to 14% of D: E wells when growing surface seawater bacterial isolates at 16°C for 2 and 3 weeks respectively—therefore, when oligotrophic conditions are employed for sponge biodiscovery, with low temperatures such as 4°C, incubation periods may need to be extended past two months to account for the extreme conditions of colder water deep-sea samples.

Aside from D: E studies, we employed a wide range of growth media with the aim of culturing diverse species. ‘The great plate count anomaly’ suggests less than 1% of bacteria are cultivable within the laboratory setting, so changing parameters such as nutrients, temperature and incubation period could help to increase diversity and the prospect of recovering novel species (Subramani and Aalbersberg 2013). Rodrigues and Carvalho (2022) were able to show that the use of different media resulted in a different percentage of cultivable marine bacteria, ranging from 2–45% of the total diversity observed with culture-independent methods, with Marine Agar generating the greatest cell numbers. Cultivation of sponge associated bacteria is generally very difficult, with less than 14% of microbial symbionts being recovered in some previous studies (Dat et al. 2021). Employing a range of conditions in our study generated a larger culture collection than if only one method had been used. Actinobacteria directed media enabled the cultivation of many *Actinobacteria*, including *Streptomyces*, renowned for their antimicrobial potential, further supporting the suggestion that these media are good candidates for antimicrobial discovery directed cultivation methods. The implementation of these nutrient conditions with the *Pheronema* sponges also allowed investigation of less commonly bioactive species like *Gordonia* and *Williamsia*, both showing anti-Gram-positive activity in initial screening efforts, as has been reported previously from marine samples (Elfalah et al. 2013, de Menezes et al. 2019). To the best of the authors’ knowledge, this is the first time *Gordonia* and *Williamsia* have been isolated from *Pheronema* sponges. Overall, using different types of media enabled cultivation of many species but for future bioactivity studies, a wide range of media targeting *Actinobacteria , Proteobacteria*, and other Gram-negative bacteria could yield a larger percentage of sponge-associated bacteria, with novel taxa being discovered, as these are generally less commonly cultured genera.

Multiple morphologically similar isolates were selected from the same plate and between plates in the various culture-based methods in order to ensure a good selection of bacteria were available for screening. It is known that bacteria of the same species can have strain and environment-specific adaptations, therefore, not biasing for morphologically distinct isolates from the beginning enabled investigation of a larger and more representative sample of the isolates present (Romano et al. 2018). However, the increased (human and material) resource implications of this approach should be acknowledged, and an extensive ‘cost-benefit’ analysis could yield valuable insights. Our approach also enabled more effective selection of isolates for 16S rRNA gene sequencing; if there were multiple morphologically similar isolates the most active isolate was chosen to be pursued for 16S rRNA gene sequence identification. On two occasions, isolates were identified as being most similar to the same database reference species but they differed significantly in morphology and bioactivity. This observation has been reported previously where the 16S rRNA gene of screened isolates is almost identical and yet there are considerable differences in secondary metabolite capabilities and expression (Antony-Babu et al. 2017, Sottorff et al. 2019). This is, in some ways, not unexpected, given the variability that may be present in carriage of BGCs in individual isolates.

Using an OSMAC approach whilst screening isolates for bioactivity resulted in the discovery of 11 isolates with anti-Gram-positive activity that would have otherwise been missed with initial screening. Of those 11, there were 7 isolates that exhibited activity in only one condition. Condition specific activity has also been evidenced in other studies. Matobole et al. (2017) implemented an early-stage OSMAC step and observed activity from isolates in only one or two conditions and similarly no condition triggered activity in all isolates. OSMAC studies are generally implemented when isolates have already been determined as active or shown to harbour cryptic BGCs within the genome. At those stages an OSMAC experiment is used in attempts to increase the proportion of active BGCs that may produce novel compounds or increase the production of NPs already being produced (Romano et al. 2018). Implementation of OSMAC approaches at earlier stages here allowed for the discovery of isolates that would otherwise have been missed. Whilst an OSMAC approach can help to increase the number of hits within the culture collection, numerous conditions can be used alone and in conjunction with one another. Employing a range of conditions in attempts to replicate the original environment or to induce stress could result in the discovery of many novel compounds but it is worth considering that, as shown with this study, activity can be very nutrient specific, so some active isolates may still be missed during screening.

Altering pH conditions between acid and alkali to impact isolate recovery has been described in previous literature reviews, but few studies have used pH adjustments to trigger bioactivity. Research into pH alterations has mixed outcomes and mostly focuses on triggering secondary metabolite production from one specific species. For example, Sarkar et al. (2010) demonstrated that *Streptomyces* sp. from the intertidal regions of *Sundarbans* delta off the Bay of Bengal, had antimicrobial production that was optimal at a pH of 9, with activity evidenced from pH 7 to pH 10, and no activity was observed at pH 4. However, *Streptomyces* sp. A1 produced streptazolins at pH 8.2 and new rubromycin metabolites at pH 5.7 (Bode et al. 2002). Similarly, *Pseudoalteromonas piscida* PG-02 was shown to have antimicrobial activity between pH 6 and pH 9, although no pH values were tested below pH 6, so it is unclear if production of this antimicrobial is alkaline dependent (Darabpour et al. 2012). Our results are consistent with Williams et al. (2020), where there were slightly more active isolates in a pH 5 condition, than at pH 9, although with both our study and that of Williams et al. (2020), there is only a small difference in the number of active hits.

## Conclusion

Here, we cultured 389 isolates from samples of *P. carpenteri* sponge using D: E and Actinomycete-directed cultivation strategies and discovered numerous active isolates. We revealed that selecting isolates based upon morphology alone could be under-representative, with potentially bioactive strains being missed. Similarly, we reveal that bioactivity can be very specific to environmental conditions and OSMAC approaches should be considered at the initial screening stage to increase the pool of active isolates for progression, rather than just revealing the ‘low hanging fruits’. Finally, we also show that the glass sponge *P. carpenteri* carries sponge-associated bacteria that have activity towards ESKAPE pathogens and we suggest that this group and other deep sea sponges warrant more detailed investigation for novel bioactive compounds of medical relevance.

## Author contributions

This study was conceptualized, planned, and conducted by all authors. Sponges were collected by K. L. Howell. Data curation, analysis, and visualizations were carried out by J. Conway. Writing of the original draft was performed by J. Conway and M. Upton, and all authors contributed to the reviewing and editing of this paper. Supervision was provided by M. Upton, K. L. Howell, G. G. January, and R. Dorrington.

## Supplementary Material

xtaf016_Supplemental_Files

## Data Availability

Bacterial 16S rRNA gene sequences have been deposited in GenBank, DNA Data Bank of Japan (DDBJ), and the European Nucleotide Archive (ENA) under the project SUB14999354. Individual 16S rRNA gene sequences can be found with BioSample accession ID’s (PQ865654–PQ865710).
