# Supplementary material for: Impact of growth conditions on the abundance and diversity of cultivable bacteria recovered from Pheronema carpenteri and investigation of their antimicrobial potential
Source: FEMS Microbes. 2025 Nov 7;6:xtaf016. doi: 10.1093/femsmc/xtaf016 (PMC12658890; doi:10.1093/femsmc/xtaf016)
Supplement: xtaf016_Supplemental_Files [file xtaf016_supplemental_files.zip › FEMSMC_2025_015_Final_Suppl_Mat.docx]

Supplementary Figure S1: Flow-diagram indicating the steps undertaken in the different approaches used to investigate the cultivable microbiota in samples of the sponge *Pheronema carpenteri.*

*
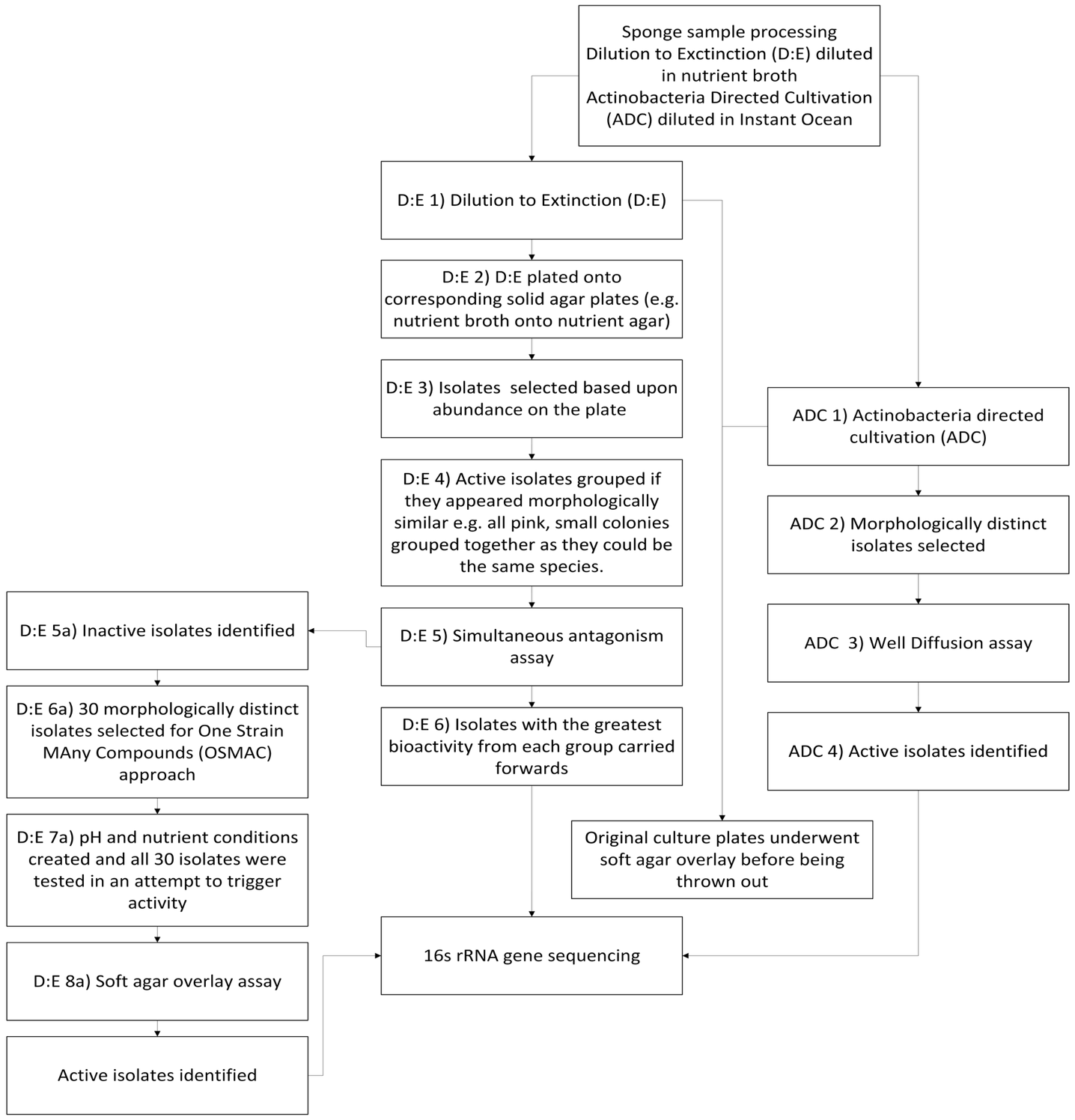
*

Supplementary Table 1: A list of all media conditions employed and the composition. All media components were sourced from Fisher, UK unless otherwise stated and all media was ordered in ready to go unless otherwise stated as ‘in-house recipe’.

| **Media/Component** | **Code** | **Composition (g/L)** | **Reference** | **Source** |
| --- | --- | --- | --- | --- |
| Marine Broth 2216 | MA | Peptone (5g/l), Yeast Extract (1g/l), Ferric Citrate (0.1g/l), Sodium Chloride (19.45g/l), Magnesium Chloride (5.9g/l), Magnesium Sulfate (3.24g/l), Calcium Chloride (1.8g/l), Potassium Chloride (0.55g/l), Sodium Bicarbonate (0.16g/l), Potassium Bromide (0.08g/l), Strontium Chloride (0.03g/l), Boric Acid (0.022g/l), Sodium Silicate (.004g/l), Sodium Fluoride (0.0024g/l), Ammonium Nitrate (0.0016g/l), Disodium Phosphate (0.008g/l) | Zobelle, (1941)  Sanz-Seaz *et al.,* (2021) | Merck |
| Reasoner’s 2A Agar | R2A | Yeast Extract (0.5g/l), Proteose Peptone (0.5g/l), Casein Hydrolysate (0.5g/l), Glucose (0.5g/l), Soluble Starch (0.5g/l), Sodium Pyruvate (0.3g/l) Dipotassium Hydrogen phosphate (0.3g/l), Magnesium Sulphate Anhydrous (0.024g/l), Agar (15.0g/l) | Van der linde *et al.,* (1999) | Merck |
| Reasoner’s 2A Broth | R2B | Casein Digest Peptone (0.25g/l), Peptic Digest of Animal Tissue (0.25g/l), Magnesium Sulfate Heptahydrate (0.5g/l) | Van der linde *et al.,* (1999) | Merck |
| Nutrient Broth | NB | Peptones (15.0g/l), Yeast Extract (3.0g/l), Sodium Chloride (6.0g/l), D(+) glucose (1.0g/l) | Fahy and Persley (1983) | Merck |
| Starch Casein Agar | SCA | Soluble Starch (10g/l), Dipotassium Phosphate (2g/l), Potassium Nitrate (2g/l)_,_ Casein (0.3 g/l), Magnesium Sulfate Heptahydrate (0.05 g/l), Calcium Carbonate (0.02g/l)_,_ Iron Sulfate Heptahydrate (0.01g/l), Agar (15g/l) | Chiaki *et al.,* (2010) | In-house recipe |
| International Streptomyces Project 2 broth | ISP2 | Yeast Extract (4.0g/l), Malt Extract (10.0g/l), Dextrose (4.0g/l), | Williams *et al.,* (2020) | In-house recipe |
| Oatmeal | OM | Oatmeal (60g/l), Agar (15g/l) | Subramani and Sipkema, (2019) | In-house recipe |
| Actinomycete Agar | ADC | Sodium Caseinate (2g/l), L-Asparagine (0.1g/l), Sodium Propionate (4g/l), Dipotassium Phosphate (0.5g/l), Magnesium Sulfate (0.1g/l), Ferrous Sulfate (0.001g/l), supplemented with glycerol (5 ml/l) | Montalov *et al.,* (2014) | In-house recipe |
| Humic Vitamin Agar | HVA | Humic Acid (1g/l), Potassium Chloride (1.7g/l), Disodium Phosphate (0.5g/l), Magnesium Sulphate (0.5g/l), Calcium Carbonate (0.02g/l), Iron Sulphate (0.01g/l), VB Stock Solution (1ml/L) | Hayakawa *et al.,* (2008) | In-house recipe |
| M9 Minimal Media | M9 | 5x M9 Salt Solution (10.44g/L)  1M Magnesium Sulphate (2mL)  1M Calcium Chloride (0.1mL)  Thiamine Mononitrate (0.1mL)  Casamino Acids (1g) | Williams *et al.,* (2020) | In-house recipe |

Supplementary Table 2: Isolates cultivated in dilution to extinction (D:E) and actinobacteria-directed cultivation (ADC) exhibiting bioactivity, displayed in the phylogenetic tree (Fig. 6). Those where N/A is in ‘bioactivity’ shows they were selected based upon morphological differences.

| **Isolate Number** | **Phylum** | **Genus** | **% Match (bp)** | **Closest NCBI Match** | **Bioactivity** |
| --- | --- | --- | --- | --- | --- |
| 3.2 | Firmicutes | *Brevibacillus* | 99% (1402) | *Brevibacillus parabrevis GF13* | N/A |
| P4 | Firmicutes | *Paenibacillus* | 100% (1434) | *Bacillus mycoides BPN57/2* | N/A |
| 4.2 | Actinobacteria | *Streptomyces* | 99% (1373) | *Streptomyces pratensis YA10* | N/A |
| 5.2 | Pseudomonadota | *Naxibacter* | 99% (1390) | *Naxibacter haematophilus Ag15* | N/A |
| 6.2 | Firmicutes | *Bacillus* | 99% (1417) | *Bacillus mycoides* | N/A |
| 8.2 | Firmicutes | *Bacillus* | 99% (1389) | *Bacillus subtilis* | N/A |
| 9 | Firmicutes | *Bacillus* | 99% (1431) | *Bacillus inaquosorum* T1 | N/A |
| 9.2 | Actinobacteria | *Gordonia* | 99% (1291) | *Gordonia polyisoprenivorans* | *S. aureus* |
| P10 | Actinobacteria | *Streptomyces* | 100% (1504) | *Streptomyces microflavus NA06532* | *M. luteus* / *S. aureus* /*Ent. faecalis* |
| 11 | Firmicutes | *Bacillus* | 99% (1428) | *Bacillus subtilis* DK15 | *S. aureus* |
| 11.2 | Actinobacteria | *Streptomyces* | 99% (1364) | *Streptomyces pratensis* | *S. aureus/ Ent. Faecalis* |
| 12.2 | Actinobacteria | *Williamsia* | 99% (1367) | *Williamsia muralis EGI_MS-37* | *S. aureus/ M. luteus* |
| 13r2 | Firmicutes | *Bacillus* | 99% (1436) | *Bacillus subtilits* Bs37 | *S. aureus/ Ent. Faecalis/* VRE |
| 13.2 | Firmicutes | *Paenibacillus* | 99% (1425) | *Paenibacillus polymyxa YC0573* | *S. aureus/VRE/ Ent. faecalis* |
| 14.2 | Firmicutes | *Paenibacillus* | 99% (1426) | *Paenibacillus polymyxa YC0573* | *S. aureus/VRE/ Ent. faecalis* |
| L15 | Firmicutes | *Bacillus* | 100% (1430) | *Bacillus subtilis subsp.* 168 | *S. aureus* |
| 15.2 | Firmicutes | *Paenibacillus* | 99% (1406) | *Paenibacillus peoriae* | N/A |
| L16 | Firmicutes | *Bacillus* | 100% (1429) | *Paenibacillus polymyxa YC0573* | *E. coli* |
| 16.2 | Firmicutes | *Bacillus* | 99% (1414) | *Bacillus altitudinis 41KF2bT.26* | N/A |
| 17.2 | Firmicutes | *Paenibacillus* | 99% (1416) | *Paenibacillus polymyxa YC0573* | *E. coli* |
| 18.2 | Firmicutes | *Paenibacillus* | 99% (1408) | *Paenibacillus polymyxa YC0573* | *E. coli* |
| 22 | Firmicutes | *Bacillus* | 99% (1437) | *Bacillus subtilis* KBL27 | *E. coli/ S. aureus* |
| 28 | Firmicutes | *Bacillus* | 99% (1431) | *Bacillus subtilis soli* G2B | *S. aureus/ Ent. faecalis* |
| 33 | Firmicutes | *Bacillus* | 99% (1424) | *Bacillus aerophilus* Z54 | *S. aureus* |
| 33.3 | Actinobacteria | *Dermacoccus* | 100% (1352) | *Dermacoccus nishinomiyaensis ZJH* | *S. aureus* / VRE |
| 36 | Actinobacteria | *Brevibacterium* | 99% (1376) | *Brevibacterium sanguinis* CB8 | *S. aureus* /VRE |
| 36.7 | Actinobacteria | *Brevibacterium* | 99% (1398) | *Brevibacterium sp. CPBF* | N/A |
| 37 | Actinobacteria | *Williamsia* | 100% (1336) | *Williamsia sp. ISE_22* | *M. luteus* |
| 38 | Actinobacteria | *Dermacoccus* | 99% (1333) | *Dermacoccus nishinomiyaensis GISt318* | *S. aureus* / *E. coli* |
| 51 | Firmicutes | *Bacillus* | 94% (1355) | *Bacillus subtilis XGL205* | *E. coli* / *S. aureus* / VRE/ *Ent. faecalis* |
| 60 | Firmicutes | *Bacillus* | 100% (1435) | *Bacillus subtilis* S123L | *S. aureus* |
| 86 | Firmicutes | *Bacillus* | 99% (1434) | *Bacillus subtilis CLC-F8* | *S. aureus/ Ent. faecalis* |
| 93 | Firmicutes | *Brevibacillus* | 99% (1417) | *Brevibacillus parabrevis BT332* | *S. aureus/ V*RE/ *Ent. Faecalis/ P. aeruginosa* |
| 94 | Firmicutes | *Bacillus* | 99% (1437) | *Bacillus subtilis subsp. 168* | *E. coli/ S. aureus/* VRE/ *Ent. faecalis* |
| 105 | Firmicutes | *Bacillus* | 100% (1430) | *Bacillus stercoris HY-38* | *S. aureus/* VRE/ *Ent. faecalis* |
| 112 | Firmicutes | *Bacillus* | 99% (1434) | *Bacterium strain BS0464* | *s. aureus* |
| 124 | Actinobacteria | *Micrococcus* | 100% (1380) | *Micrococcus luteus* | *M. luteus* |
| 125 | Firmicutes | *Bacillus* | 100% (1435) | *Bacillus subtilis MN227490.1* | *E. coli/ S. aureus*/ VRE |
| 126 | Firmicutes | *Staphylococcus* | 99% (1434) | *Staphylococcus saprophyticus UTI-045* | *M. luteus* |
| 130 | Firmicutes | *Paenibacillus* | 99% (1439) | *Paenibacillus polymyxa YC0573* | *E. coli/ A. baumanii* / *K. pneumoniae* / *P. aeruginosa* |
| 134 | Firmicutes | *Bacillus* | 99% (1435) | *Bacillus subtilis JCM 1465* | *S. aureus/* VRE/ *Ent. faecalis* |
| 148 | Firmicutes | *Bacillus* | 99% (1434) | *Bacillus subtilis UYY* | *S. aureus/ E. coli/ A. baumanii* / *K. pneumoniae* / *P. aeruginosa* |
| 152 | Firmicutes | *Bacillus* | 100% (1437) | *Bacillus subtilis KBL27* | *S. aureus* /VRE/ *E. coli* |
| 153 | Firmicutes | *Bacillus* | 100% (1436) | *Bacillus subtilis SR3-30* | *S. aureus* /VRE |
| 157 | Firmicutes | *Bacillus* | 100% (1436) | *Bacillus subtilis S12L* | *S. aureus* /VRE |
| 179 | Firmicutes | *Bacillus* | 100% (1433) | *Bacillus subtilis* | *S. aureus* */E. coli*/VRE/ *Ent. faecalis* |
| 186 | Firmicutes | *Bacillus* | 100% (1431) | *Bacillus subtilis D35* | *S. aureus* |
| 201 | Firmicutes | *Paenibacillus* | 99% (1437) | *Paenibacillus polymyxa YC0573* | *E. coli/ A. baumanii* / *K. pneumoniae* |
| 220 | Firmicutes | *Bacillus* | 99% (1436) | *Bacillus subtilis stercoris* | *S. aureus/ Ent. faecalis* |
| 229 | Firmicutes | *Paenibacillus* | 100% (1430) | *Paenibacillus polymyxa* | *E. coli/ A. baumanii* / *K. pneumoniae* / *P. aeruginosa* |
| 259 | Actinobacteria | *Streptomyces* | 99% (1391) | *Streptomyces sp. YH4* | *S. aureus* |
| 262 | Firmicutes | *Paenibacillus* | 99% (1441) | *Paenibacillus peoriae* | *S. aureus/ E. coli/ A. baumanii* / *K. pneumoniae* / *P. aeruginosa* |
| 278 | Firmicutes | *Bacillus* | 100% (1437) | *Bacillus subtilis KBL27* | *S. aureus/ E. coli* |
| 316 | Firmicutes | *Bacillus* | 99% (1439) | *Bacillus subtilis KBL27* | *S. aureus* |
| 321 | Firmicutes | *Brevibacillus* | 100% (1417) | *Brevibacillus parabrevis* | *S. aureus/ Ent. faecalis* |
| 322 | Firmicutes | *Bacillus* | 99% (1436) | *Bacillus subtilis* | *E. coli* / *S. aureus* |

Media References:

Chiaki, I., Naoko, K., Masazumi, K,. Takeshi, K., Naoko, H. 2007. Actinomycetologica, 21:27- 31.

Fahy, P.C., Persley, G.J., Plant Bacterial Diseases. A Diagnostic Guide [FAHY, P.C. and G.J. PERSLEY (editors)](https://www.abebooks.co.uk/book-search/author/fahy-p-c-g-j-persley/), Published by Academic Press, Sydney, 1983ISBN1224766032, 9781224766033

Hayakawa, M. (2008). Studies on the Isolation and Distribution of Rare Actinomycetes in Soil. *Actinomycetologica*, *22*(1). https://doi.org/10.3209/saj.saj220103

Montalvo, N. F., Davis, J., Vicente, J., Pittiglio, R., Ravel, J., & Hill, R. T. (2014). Integration of culture-based and molecular analysis of a complex sponge-associated bacterial community. *PLoS ONE*, *9*(3).

Subramani, R., & Sipkema, D. (2019). Marine rare actinomycetes: A promising source of structurally diverse and unique novel natural products. In *Marine Drugs* (Vol. 17, Issue 5).

van der Linde, K., Lim, B. T., Rondeel, J. M. M., Antonissen, L. P. M. T., & de Jong, G. M. T. (1999). Improved bacteriological surveillance of haemodialysis fluids: A comparison between Tryptic soy agar and Reasoner’s 2A media. *Nephrology Dialysis Transplantation*, *14*(10). https://doi.org/10.1093/ndt/14.10.2433

Williams, S. E., Stennett, H. L., Back, C. R., Tiwari, K., Gomez, J. O., Challand, M. R., Hendry, K. R., Spencer, J., Essex-Lopresti, A. E., Willis, C. L., Curnow, P., & Race, P. R. (2020). The Bristol sponge microbiome collection: A unique repository of deep-sea microorganisms and associated natural products. *Antibiotics*, *9*(8).

Zobell, Claude E.. 1941. "Studies on marine bacteria. I. The cultural requirements of heterotrophic aerobes." *Journal of Marine Research* 4, (1). https://elischolar.library.yale.edu/journal_of_marine_research/582
