# Supplementary material for: Impact of growth conditions on the abundance and diversity of cultivable bacteria recovered from Pheronema carpenteri and investigation of their antimicrobial potential
Source: FEMS Microbes. 2025 Nov 7;6:xtaf016. doi: 10.1093/femsmc/xtaf016 (PMC12658890; doi:10.1093/femsmc/xtaf016)
Supplement: xtaf016_Supplemental_Files [file xtaf016_supplemental_files.zip › FEMSMC-2025-015.R2 one sentence summary.docx]

Bacteria that produce potentially new antibiotic compounds can be found in deep sea sponges like Pheronema carpenteri using a range of microbial growth conditions
