# Supplementary material for: Impact of growth conditions on the abundance and diversity of cultivable bacteria recovered from Pheronema carpenteri and investigation of their antimicrobial potential
Source: FEMS Microbes. 2025 Nov 7;6:xtaf016. doi: 10.1093/femsmc/xtaf016 (PMC12658890; doi:10.1093/femsmc/xtaf016)
Supplement: xtaf016_Supplemental_Files [file xtaf016_supplemental_files.zip › FEMSMC-2025-015.R2 supplementary data - reviews and author response.docx]

**Reviews of manuscript FEMSMC-2025-015**

**Reviewer 1**

**Recommendation: Minor Revision**

This is an interesting study exploring the antimicrobial potential of the microbiota of hexactinellid glass sponges. Glass sponges, almost exclusive to the deep-sea, are understudied compared to demosponges, which adds to the novelty of the research. The combination of both cultivation and bioactivity testing represents a substantial body of work. However, presentation of methods and results is at times confusing and inconsistent. The following comments should improve clarity, reproducibility and consistency.

Major points

1. Clarity of Methods and Workflow

- The description of isolate selection, grouping and downstream assays (pp. 234–239 and other areas) is hard to follow. A concise flow diagram delineating each step —from initial activity screen, through grouping, to re-screening—would clarify the experimental pipeline. This is particularly important due to the various techniques employed in the study, as it is easy for the reader to get lost.

- Coverage of well is determined by visual inspection, yet the % values used are very specific E.g. 3%, 28-68%. Is this really possible? This might be hard to replicate

- A species level assignment of the sponges on ‘morphological’ inspection lacks detail. Is this a spicule inspection? More details

- Please ensure reagent sourcing and nomenclature is consistent (e.g. “Fisher Scientific, UK” vs. “Fisher”).

2. Data Presentation

- Figures 2 and 4 use inconsistent sponge‐ID formats; adopt one convention throughout.

- Figure 3 panels could be reorganized (e.g. box around ADC panels B + C) to highlight experimental comparisons. Helping the reader see the different isolation workflows more intuitively

3. Phylogenetic Analysis

- The 16S rRNA gene phylogenetic tree should be rooted with an appropriate outgroup (e.g. E. coli or other).

- Highlighting conditions by red font within the tree is visually confusing—consider alternative encodings rather than forcing the tree into two sections (e.g. symbols, text colour).

4. Discussion

- A comment on antimicrobial compounds produced by Bacillus that may have been responsible for the activity observed. There are common compounds in the literature that could be mentioned

- A few sentences on differences between sponges would be of interest to the reader. Was there a large difference in taxonomy recovered from the 3 samples of Pheronema carpenter? Would you expect them to be similar?

Minor corrections:

Introduction

37 – Do under-explored environments reduce cost? Rephrase to clarify that rediscovery of known compounds is costly, and under-explored environments may reduce this risk.

127 – The sponges JC136_125 and 136 are very spatially close to each other. Did we see similarity in the isolated bacteria? Could this be mentioned in the discussion if so?

133 – Delete redundant sentence

141 – ‘Actinobacteria’ Phyla should not be italics*

*Please correct elsewhere in the manuscript

171 – capital not needed for Sodium.

172 – Fisher Scientific- UK?

179 – When first introduced, spell out genus (Staphylococcus, Escherichia).

184 – The temperature shouldn’t be in brackets here and should have space between number and °C

195 –Nystatin, Nalidixic acid and Cycloheximide can be lower case

208 – 600nm should be subscript

215 – In triplicate rather than (n=3)

234-237 – I think I understand what you did but this paragraph is a little unclear please rewrite for clarification

239 – This section is quite unclear. So all active isolates were taken forward but some were grouped together? I think this section needs to be shorten and revised. There a numerous assays at numerous points and it can be difficult to explain the whole thing. – a flow diagram in the methods would help the reader understand.

265 – I believe ‘silent’ is more accurate

281 – Cite tools if available (E.g. iTOL, blastN) and give versions.

Results

299 – remove ‘it appeared that’

302 - Replace “full strength nutrients” with a clearer term (e.g. “undiluted medium”)

304 – I think these values are too precise for measure by eye. Can we really measure 28% coverage? Or 3%?

310 – Omit first sentence

354 – Figure 3. Average diversity in various nutrient strengths? Please clarify

362 – 273 + 116 isolates = 389 total; reconcile with abstract.

368–369: Rephrase for clarity—explain the relationship between isolate counts and activity rates.

378 – Derfine media acronym ACT, ensure all media are introduced in methods. MA has the highest number of isolates but then only has one active isolate from each media? This appears to be ADC vs DE but is not very clear.

427 – Most Bacillus isolates are B. subtilis; please state this observation.

434 – As above, reroot tree and adjust highlighting scheme

443 – rDNA is not really used. 16S rRNA gene sequence would be more accurate

438 – Consider whether observed activity differences stem from isolation versus assay media.

472 – Replace vague “no observed impact” with “no isolates showed activity on R2A.”

484 – Consider use of colour or shading in tables to improve readability.

Discussion

492 – Emphasize novelty from this study over Koch et al. 2021 findings

510 – Discuss absence of Pseudovibrio isolates—possible explanations (media bias, host specificity).

541 – Clarify whether referring to “greatest cell numbers” or “highest recovered diversity.”

608 – 389 isolates. The abstract states 386 bacterial isolates. Please amend

614 – ‘low hanging fruits’ (needs a second ‘)

**Reviewer 2**

**Recommendation: Minor Revision**

“Impact of growth conditions on the abundance and diversity of cultivable bacteria recovered from Pheronema carpenteri and investigation of their antimicrobial potential”

The paper outlines the isolation of isolates from the sponge, Pheronema carpenteri, and uses various growth and OSMAC protocols to increase the recovery of diverse bacterial species and trigger cryptic antibiotic pathways. It is clear a considerable amount of work has been undertaken and there is some interesting data that would be of value to the wider readership in the development of antimicrobial discovery. Generally, the manuscript is well written and does not overstretch its claims based on the data, but I have a few minor comments and queries below that I would like addressed.

Reviewer comments

The citation order is inconsistent when multiple citations are used. Some are in alphabetical order (e.g., line 39, Srinivasan et al., 2021; Trenozhnikova and Azizan, 2018) whereas others are in chronological order (e.g., line 48, Varijakzhan et al., 2021; Hong et al., 2022; Khodzori et al., 2023). Please review these be consistent, using the journal’s preferred ordering system.

Line 48: Citation Hong et al 2022 is not in the reference list. Is this meant to be the Kong et al 2022 reference or another paper that is not in the list?

Line 145-146: Marine broth is used in the initial experiment, but it is abbreviated to MA (which suggests Marine Agar) rather than MB (Marine Broth). The other liquid media in this section are referred to as broths (e.g., Nutrient Broth is NB). Given the liquid culture in 96-well plates is initially used and then transferred to reciprocal solid media, this does get a little confusing when reading through and correcting this would help the reader to know which stage the work is at.

Line 157: NB is given as an example so it should be given an ‘e.g.,’ (e.g., NB inoculated onto Nutrient Agar etc.)

Line 179: What culture collection is S. aureus 48491 from? I had a quick look, but it was not immediately obvious where it is from. For anyone wishing to get this strain to replicate this work, this is needed.

Line 183-184: It outlines that the overnight broths were adjusted to 0.05 OD600/ml and used to seed 0.75% soft agar but is it unclear how much of the adjusted broth was used and in to how much soft agar overlay. For anyone wishing to replicate this, those details would be helpful.

Line 190: The citation is Simeis and Sarra 2021, but the correct citation is De Simesis and Sarra 2021 to match the reference.

Line 199: Can the authors confirm that the plates were incubated aerobically as it is not stated, and various environmental conditions exist.

Line 212: How was the in-house C. albicans isolate confirmed to be C. albicans?

Line 228: There needs to be a space between ‘at’ and ‘12,000x g’

Line 272: Barns et al 1991 is cited here, however it does not appear in the reference list. Do the authors mean Weisburg et al 1919, which does appear in the reference list?

Line 318: Contractions are not used in scientific writing, so please change ‘didn’t’ to ‘did not’.

Line 320: This part is referring to growth in the wells but refers to NA which is Nutrient Agar rather than NB which is Nutrient Broth. This follows on from my comment on lines

145-146, with it being a little tricky to work out whether it is solid are liquid media. Please read through and correct were necessary so the right media type is mentioned.

Line 346: There is reference to ISP2 30% here, and in figure 3 each media has 30% after it, but I am not clear what the 30% is referring to. In the material and methods under the Actinobacteria Directed Cultivation section, I see reference to Instant Ocean added at 3.3%w/v (line 192) but do not see any reference to 30% throughout the methods. The next sentence (line 346) does mention Instant Ocean but it is not at 30%, so can the authors please clarify what the 30% is referring to?

Line 346-348: It states that the presence of Instant Ocean seemed to decrease the diversity present on the plate in all cases except for OAT where the was a slight increase in diversity (fig 3B). When I look at fig 3B, I see a slight increase in diversity in OAT with Instant Ocean compared to OAT alone, but also note an increase in HVA plus Instant Ocean compared to HVA alone. Why is this not mentioned here?

Line 356: The legend refers to conditions with and without seawater, but is this Instant Ocean rather than seawater? If it is seawater, then I do not see any mention of this under the Actinobacteria Directed Cultivation section, only Instant Ocean. (The only mention of seawater in the methods is on line 120 during the initial collection phase).

Line 381: It mentions MA for the D:E conditions, but is this the broth media so should be MB? I feel the broth incubation will have had a big impact on the culturing of the diversity which is then plated on to the agar. This corresponds to previous comments made about this above.

Lines 384-385: Would the journal prefer the word versions of the numbers rather than the numerical version? Typically, below ten, the word is used.

Line 509: Ton That Huu and colleagues (2021) does not appear to be in the reference list. Is this referring to Dat et al (2021) because it is not immediately clear if so, as the reference only provides the initials TTH. Please change to Dat et al (2021).

Line 543: Is this referring to Dat et al 2021 here? Please correct so it matches the reference as it is given.

Line 708: Hesketh-Best et al 2023 is not cited within the manuscript, please remove this reference or cite the reference in the appropriate place.

Figures 3 and 5: The media with the 30% (or 3.3%) is in a different order to the media without the 30% (or 3.3%). Specifically, ISP2 30% should come before OAT 30% to keep it consistent.

Figures 3 and 5: Why was SCA 30% not tested when all other media has a 30% variant included?

Figure 6: I printed out this figure and the differences in the shades between the same colour is not very prominent. Could they be made a little more distinct?

Table 2: The activity on different media at pH5 and pH7 can been seen, but at pH7 I do not see the various media. Were the isolates on the pH7 media active or not?

**Associate Editor Recommendation: Minor Revision**

**EIC Decision: Major Revision**

**Author's Response to Decision Letter for (FEMSMC-2025-015)**

We would like to take the opportunity to thank the editor and reviewers for the time taken to review this manuscript and for the insightful and constructive nature of the comments. In addressing these comments, we feel that the manuscript has been improved. We hope the responses to the comments are clear and address the points raised effectively. We look forward to your feedback.

Responses to each comment are provided below:

Reviewers' Comments to Authors:

Reviewer: 1

Comments to the Author

This is an interesting study exploring the antimicrobial potential of the microbiota of hexactinellid glass sponges. Glass sponges, almost exclusive to the deep-sea, are understudied compared to demosponges, which adds to the novelty of the research. The combination of both cultivation and bioactivity testing represents a substantial body of work. However, presentation of methods and results is at times confusing and inconsistent. The following comments should improve clarity, reproducibility and consistency.

Major points

1. Clarity of Methods and Workflow

- The description of isolate selection, grouping and downstream assays (pp. 234–239 and other areas) is hard to follow. A concise flow diagram delineating each step —from initial activity screen, through grouping, to re-screening—would clarify the experimental pipeline. This is particularly important due to the various techniques employed in the study, as it is easy for the reader to get lost.

RESPONSE - We thank the reviewer for this suggestion. We have created a flow diagram and added it into the supplementary section, with reference to each of the various methodological steps used within the manuscript. Steps involving Actinobacteria Directed Cultivation (ADC) and Dilution to Extinction (D:E) are outlined, e.g. Supplementary Figure 1: ADC step 1. This is to improve the flow of the manuscript and enable the reader to visualise the order of each step.

- Coverage of well is determined by visual inspection, yet the % values used are very specific E.g. 3%, 28-68%. Is this really possible? This might be hard to replicate.

RESPONSE - We agree with this comment and would like to explain that the percentage values reported were based on visual inspection and estimated in increments of 10% and an average taken of three repeats. We agree that the previous wording did not communicate this clearly and we see how this could lead to misunderstanding. We have revised the text to clarify that the values are estimated to the nearest 10% based on visual inspection, conducted in triplicate to improve consistency. We have also updated the reported ranges (e.g., 28–68%) to reflect this estimation method more transparently (e.g., “~30–70%”).

- A species level assignment of the sponges on ‘morphological’ inspection lacks detail. Is this a spicule inspection? More details

RESPONSE - We have included the following in the manuscript “Internal and external morphological observations (body shape, size, spicules) were undertaken to assign them as Pheronema carpenteri sponges as outlined by Hesketh-Best et al., (2019).” In the Hesketh-Best et al., (2019) study the authors classified the sponge samples used in our current work and therefore they have been cited in the manuscript for further information.

- Please ensure reagent sourcing and nomenclature is consistent (e.g. “Fisher Scientific, UK” vs. “Fisher”).

RESPONSE - Thank you for highlighting this, we have made the necessary adjustments throughout the manuscript.

2. Data Presentation

- Figures 2 and 4 use inconsistent sponge‐ID formats; adopt one convention throughout.

RESPONSE - Thank you for highlighting this, all figures have been checked and amended to one standard ID format (JC136_066, JC136_125 and JC136_134).

- Figure 3 panels could be reorganized (e.g. box around ADC panels B + C) to highlight experimental comparisons. Helping the reader see the different isolation workflows more intuitively.

RESPONSE - Boxes have been included to highlight the various cultivation techniques, ADC cultivation is now shown through black boxes surrounding the figures, and this is explained in the legend for Figure 3A.

3. Phylogenetic Analysis

- The 16S rRNA gene phylogenetic tree should be rooted with an appropriate outgroup (e.g. E. coli or other).

- Highlighting conditions by red font within the tree is visually confusing—consider alternative encodings rather than forcing the tree into two sections (e.g. symbols, text colour).

RESPONSE - Thank you for this recommendation, the tree has been re-run and re-rooted with an appropriate outgroup Bacteriodes fragilis NCTC 9343. Secondly, assignment of cultivation methods has been shown using red for ADC and blue squares for D:E, we believe this looks less visually confusing.

4. Discussion

- A comment on antimicrobial compounds produced by Bacillus that may have been responsible for the activity observed. There are common compounds in the literature that could be mentioned

RESPONSE - Additional paragraphs have been added to the discussion involving the various compounds produced by Bacillus: ‘Similarly to our observations, previously studies have reported Bacillus species to harbour antimicrobial capabilities. Activity towards Gram-negative species has been observed from Bacillus subtilis such as polymyxin, difficidin and bacitracin previously (Santhi et al., 2017). Similarly, bioactive Bacillus sp. have been recovered from the sponge Dysidea avara (Li et al., 2007). Bacillus sp. isolated from sponge class Homoscleromorpha showed evidence of having activity towards multidrug resistant strains and these could be an additional source of bioactive compounds (Freitas-Silva et al., 2020). The presence of a large number of bioactive Bacillus isolates in the current study is promising and warrants further investigation of Demosponges and members of other sponge classes.’

Minor corrections:

Introduction

37 – Do under-explored environments reduce cost? Rephrase to clarify that rediscovery of known compounds is costly, and under-explored environments may reduce this risk.

RESPONSE - Re-phrased to ‘However, the high rate of rediscovery and the significant costs associated with their development requires alternative environments and strategies to be used to reduce the risk of rediscovery (Devine, Hutchings and Holmes, 2017)’

127 – The sponges JC136_125 and 136 are very spatially close to each other. Did we see similarity in the isolated bacteria? Could this be mentioned in the discussion if so?

RESPONSE - The reviewer raises an interesting point. It is not possible to provide a conclusive response based on the data presented here, given that the strain identification work was based on a sub-set of isolates. In a linked study, submitted separately, we have conducted 16S rRNA profiling of the same samples described in this study. In the profiling data, it is apparent that samples JC136_125 and JC136_134 have some overlaps in microbiome, but the dominant phyla are actually different. These observations of intraspecies differences are consistent with similar reports for other marine sponges. Whilst this is not a full answer to the reviewer’s comment, we trust that it is a sufficient response for now.

133 – Delete redundant sentence

RESPONSE - Done

141 – ‘Actinobacteria’ Phyla should not be italics*

*Please correct elsewhere in the manuscript

RESPONSE - Thank you, this has been amended throughout the manuscript

171 – capital not needed for Sodium.

RESPONSE - This has been corrected, thank you for bringing this to our attention.

172 – Fisher Scientific- UK?

RESPONSE - This has now been corrected throughout.

179 – When first introduced, spell out genus (Staphylococcus, Escherichia).

RESPONSE - Done

184 – The temperature shouldn’t be in brackets here and should have space between number and °C

RESPONSE - This has now been corrected throughout the whole manuscript.

195 –Nystatin, Nalidixic acid and Cycloheximide can be lower case

RESPONSE - Thank you, amended

208 – 600nm should be subscript

RESPONSE - Done

215 – In triplicate rather than (n=3)

RESPONSE - Done

234-237 – I think I understand what you did but this paragraph is a little unclear please rewrite for clarification

RESPONSE - This has been rephrased to the following “To ensure all isolates had been assayed for bioactivity, all culture plates from D:E and ADC cultivation studies, were tested for activity with a soft agar overlay, using M. luteus at OD600nm of 0.1, to ensure all colonies had been tested for activity before discarding plates.”

239 – This section is quite unclear. So all active isolates were taken forward but some were grouped together? I think this section needs to be shorten and revised. There a numerous assays at numerous points and it can be difficult to explain the whole thing. – a flow diagram in the methods would help the reader understand.

RESPONSE - Thank you for this, this has also been outlined in the flow diagram to give clarity and rephrased in the manuscript to: Isolates in D:E methods were not selected based upon being morphologically distinct, therefore there was the risk that many of the isolates could be the same species. However, as this study was more focussed upon discovering bioactivity, isolates that looked morphologically similar were grouped and assayed for bioactivity. Isolates from that group with the greatest bioactivity (largest zone sizes, or activity towards Gram-negative pathogens), were then carried forwards for further study (D:E step 6).

265 – I believe ‘silent’ is more accurate

RESPONSE - Corrected

281 – Cite tools if available (E.g. iTOL, blastN) and give versions.

RESPONSE - Thank you, iTOL has version added but blastN was used on a web browser, so there was no need to download a particular version. We trust this is acceptable.

Results

299 – remove ‘it appeared that’

RESPONSE - Done

302 - Replace “full strength nutrients” with a clearer term (e.g. “undiluted medium”)

RESPONSE - Amended throughout the manuscript for consistency

304 – I think these values are too precise for measure by eye. Can we really measure 28% coverage? Or 3%? Addressed at the beginning

RESPONSE - This has been explained above.

310 – Omit first sentence

RESPONSE - This has now been removed.

354 – Figure 3. Average diversity in various nutrient strengths? Please clarify.

RESPONSE - Morphologically distinct isolates were counted across the inoculated plates and an average was taken, e.g. corresponding wells were inoculated onto agar plates and these were left to grow. At the end of the incubation period all morphologically distinct isolates were counted and an average taken on the same agar (e.g. triplicate agar plates on nutrient agar, made an average of 3 diverse colonies per plate).

362 – 273 + 116 isolates = 389 total; reconcile with abstract.

RESPONSE - Thank you this has been double checked with the raw data and amended.

368–369: Rephrase for clarity—explain the relationship between isolate counts and activity rates.

RESPONSE - This entire paragraph has now been edited for clarity and separated out: Comparison between D:E and ADC techniques cannot be made due to the nature of isolate selection and bioassay testing. D:E isolates were chosen based on the relative abundance observed on the isolation plate, not considering whether isolates were morphologically distinct, whereas ADC isolates were selected if morphologically distinct, so no conclusion as to the best method for active isolates can be drawn.

In total, 273 marine sponge isolates were recovered from three deep sea Pheronema carpenteri sponges, under various D:E conditions. Of these, most isolates were from sponge JC136_125 followed by JC136_066 and finally sponge JC136_134, with 108, 98 and 67 isolates recovered from each, respectively (Fig. 4). Of those isolates, 31% were active from sponge JC136_125, 12% from JC136_066 and 28% from sponge JC136_134.

A further 116 morphologically distinct isolates were cultured using ADC agar with active isolates generally less frequent with 9% (10) of isolates active in total across the three sponges.

We hope this is a clearer explanation for why more isolates were selected and a higher proportion of activity was observed in D:E in comparison to ADC, indicating that D:E isolates were selected and tested for bioactivity regardless of whether they looked morphologically distinct or not.

378 – Define media acronym ACT, ensure all media are introduced in methods.

RESPONSE - This has been addressed in ‘Actinobacteria Directed Cultivation using Solid Agar’, where there was a typo of ADC instead of ACT in the media list.

MA has the highest number of isolates but then only has one active isolate from each media? This appears to be ADC vs DE but is not very clear.

RESPONSE - This observation is correct - it is possible that the MA could have selected for general marine bacteria but those that grew did not show bioactivity towards the pathogens screened, with only one isolate showing bioactivity in ADC conditions with MA. Throughout the manuscript, both ADC and D:E is stated, however there is no comparisons made between the methods. As mentioned, the techniques employed were very different throughout so comparisons should not be made between the two techniques.

427 – Most Bacillus isolates are B. subtilis; please state this observation.

RESPONSE - Line 458 now reads “With the exception of a few strains identified as related to ‘Bacillus species’, Bacillus isolates were identified as Bacillus subtilis in D:E conditions, showing dominance for one species in particular.”

434 – As above, reroot tree and adjust highlighting scheme

RESPONSE - This amendment has been made and outlined above in further detail.

443 – rDNA is not really used. 16S rRNA gene sequence would be more accurate

RESPONSE - Thank you, this has been amended

438 – Consider whether observed activity differences stem from isolation versus assay media.

RESPONSE - Looking at the raw data, all isolates from Table 1 were isolated from D:E conditions, therefore discussion of isolation vs assay media is not possible, hence the differences discussed here relate to the use of various media.

472 – Replace vague “no observed impact” with “no isolates showed activity on R2A.”

RESPONSE - This now reads “Interestingly, no isolates showed activity on R2A undiluted or at the 1:10 dilution, regardless of pH.”

484 – Consider use of colour or shading in tables to improve readability.

RESPONSE - We would prefer not to do this, but will be willing to hear guidance from the Editor

Discussion

492 – Emphasize novelty from this study over Koch et al. 2021 findings

RESPONSE - The following has been added to the discussion: “Within our study the focus was upon incorporating oligotrophic (diluted) conditions to explore whether this could encourage the growth of diverse species, whereas Koch and colleagues (2021) explored the use of various medias supplemented with and without carnitine and the effects of pressure.”

510 – Discuss absence of Pseudovibrio isolates—possible explanations (media bias, host specificity).

RESPONSE - The following has been added to the discussion: “One potential explanation for the lack of Pseudovibrio in our study could be the choice of media selected; although Pseudovibrio species thrive in oligotrophic conditions, they may have been outcompeted by the dominant species e.g. Actinobacteria or Bacillus.”

541 – Clarify whether referring to “greatest cell numbers” or “highest recovered diversity.”

RESPONSE - This is indeed greatest number of cells, the paper referred to (Rodrigues and Carvalho) compares the number of cells observed under microscopy and the number that actually grew on the Marine Agar plate, and determined that Marine Agar grew 45% of the cells observed initially.

608 – 389 isolates. The abstract states 386 bacterial isolates. Please amend

RESPONSE - Amended as mentioned above

614 – ‘low hanging fruits’ (needs a second ‘)

RESPONSE - Amended as needed – thank you for this comment.

Reviewer: 2

Comments to the Author

“Impact of growth conditions on the abundance and diversity of cultivable bacteria recovered from Pheronema carpenteri and investigation of their antimicrobial potential” The paper outlines the isolation of isolates from the sponge, Pheronema carpenteri, and uses various growth and OSMAC protocols to increase the recovery of diverse bacterial species and trigger cryptic antibiotic pathways. It is clear a considerable amount of work has been undertaken and there is some interesting data that would be of value to the wider readership in the development of antimicrobial discovery. Generally, the manuscript is well written and does not overstretch its claims based on the data, but I have a few minor comments and queries below that I would like addressed.

Reviewer comments

The citation order is inconsistent when multiple citations are used. Some are in alphabetical order (e.g., line 39, Srinivasan et al., 2021; Trenozhnikova and Azizan, 2018) whereas others are in chronological order (e.g., line 48, Varijakzhan et al., 2021; Hong et al., 2022; Khodzori et al., 2023). Please review these be consistent, using the journal’s preferred ordering system.

RESPONSE - Thank you, this has now been amended to chronological order, with the oldest paper first.

Line 48: Citation Hong et al 2022 is not in the reference list. Is this meant to be the Kong et al 2022 reference or another paper that is not in the list?

RESPONSE - The following reference has been added to the list: Hong, L.-L., Ding, Y.-F., Zhang, W., & Lin, H.-W. (2022). Chemical and biological diversity of new natural products from marine sponges: a review (2009–2018). Marine Life Science & Technology, 4(3).

Line 145-146: Marine broth is used in the initial experiment, but it is abbreviated to MA (which suggests Marine Agar) rather than MB (Marine Broth). The other liquid media in this section are referred to as broths (e.g., Nutrient Broth is NB). Given the liquid culture in 96-well plates is initially used and then transferred to reciprocal solid media, this does get a little confusing when reading through and correcting this would help the reader to know which stage the work is at.

RESPONSE - Thank you very much for this observation - the relevant broths have been changed e.g. Marine broth is now MB, R2B etc.

Line 157: NB is given as an example so it should be given an ‘e.g.,’ (e.g., NB inoculated onto Nutrient Agar etc.)

RESPONSE - This has now been amended thank you for this comment.

Line 179: What culture collection is S. aureus 48491 from? I had a quick look, but it was not immediately obvious where it is from. For anyone wishing to get this strain to replicate this work, this is needed.

RESPONSE - Thank you for this comment, we have identified this particular strain using 16S rRNA gene sequencing. This isolate is an in-house strain that is generally representative of the species, with respect to antibiotic susceptibility etc. Although other S. aureus strains are available, if groups require access to this specific strain, we can provide it to them.

Line 183-184: It outlines that the overnight broths were adjusted to 0.05 OD600/ml and used to seed 0.75% soft agar but is it unclear how much of the adjusted broth was used and in to how much soft agar overlay. For anyone wishing to replicate this, those details would be helpful.

RESPONSE - Thank you for this comment, there is now a section within there that states “These overnight broths were adjusted to 0.05OD600/mL and were used to seed 0.75% soft agar that was cooled to below 50 oC and 20mL was poured onto the OSMAC plates. All plates were left to rest at room temperature for 30 minutes before being placed in an incubator overnight at 37 C (D:E step 6a).”

Line 190: The citation is Simeis and Sarra 2021, but the correct citation is De Simesis and Sarra 2021 to match the reference.

RESPONSE - This has now been changed to (De Simesis and Serra, 2021).

Line 199: Can the authors confirm that the plates were incubated aerobically as it is not stated, and various environmental conditions exist.

RESPONSE - This has now been added into the text, thank you.

Line 212: How was the in-house C. albicans isolate confirmed to be C. albicans? RESPONSE - This strain is from an in-house collection and was identified using standard phenotypic methods (e.g. biochemical tests and the germ-tube assay).

Line 228: There needs to be a space between ‘at’ and ‘12,000x g’

RESPONSE - Thank you, this has now been amended.

Line 272: Barns et al 1991 is cited here, however it does not appear in the reference list. Do the authors mean Weisburg et al 1919, which does appear in the reference list?

RESPONSE - Our apologies, this was i n the reference list but should have been cited as Weisburg not Burns (Second author), this has been amended and updated in the text – thank you for highlighting.

Line 318: Contractions are not used in scientific writing, so please change ‘didn’t’ to ‘did not’.

RESPONSE - Thank you, this has now been changed to did not and checked throughout the entire manuscript.

Line 320: This part is referring to growth in the wells but refers to NA which is Nutrient Agar rather than NB which is Nutrient Broth. This follows on from my comment on lines 145-146, with it being a little tricky to work out whether it is solid are liquid media. Please read through and correct were necessary so the right media type is mentioned.

RESPONSE - This has now been corrected throughout the manuscript and should not cause confusion again.

Line 346: There is reference to ISP2 30% here, and in figure 3 each media has 30% after it, but I am not clear what the 30% is referring to. In the material and methods under the Actinobacteria Directed Cultivation section, I see reference to Instant Ocean added at 3.3%w/v (line 192) but do not see any reference to 30% throughout the methods. The next sentence (line 346) does mention Instant Ocean but it is not at 30%, so can the authors please clarify what the 30% is referring to?

RESPONSE - Thank you, throughout the manuscript the 30% has been changed to 3.3% to correlate with the methods section.

Line 346-348: It states that the presence of Instant Ocean seemed to decrease the diversity present on the plate in all cases except for OAT where the was a slight increase in diversity (fig 3B). When I look at fig 3B, I see a slight increase in diversity in OAT with Instant Ocean compared to OAT alone, but also note an increase in HVA plus Instant Ocean compared to HVA alone. Why is this not mentioned here?

RESPONSE - HVA has now been added into this section: “The presence of 3.3% IO seemed to decrease the diversity present on the plate in all cases except for OAT and HVA where there was a slight increase in diversity (Fig. 3B).”

Line 356: The legend refers to conditions with and without seawater, but is this Instant Ocean rather than seawater? If it is seawater, then I do not see any mention of this under the Actinobacteria Directed Cultivation section, only Instant Ocean. (The only mention of seawater in the methods is on line 120 during the initial collection phase).

RESPONSE - Instant Ocean has now replaced all mentions of seawater, as Instant Ocean was used to replicate seawater throughout – apologies for the confusion caused.

Line 381: It mentions MA for the D:E conditions, but is this the broth media so should be MB? I feel the broth incubation will have had a big impact on the culturing of the diversity which is then plated on to the agar. This corresponds to previous comments made about this above.

RESPONSE - Thank you for this comment. In D:E conditions, cultures were grown for a number of weeks in various broths, diluted and undiluted. After this incubation period ended, the subsequent broths were inoculated onto the same corresponding agar (nutrient broth onto nutrient agar etc.) These were then able to incubate and observations such as abundance and diversity were made. The isolates were then picked and grown axenically before being tested for activity. Therefore, the ‘Impact of Nutrient Conditions on Active Isolate Recovery’ is referring to what was observed on the solid agar of D:E. We have added ‘After incubation on solid agar and subsequent bioactivity testing.’ As a means of hopefully clarifying this for the reader, we have added a flow diagram in the supplementary with reference throughout to improve readability.

Lines 384-385: Would the journal prefer the word versions of the numbers rather than the numerical version? Typically, below ten, the word is used.

RESPONSE - Thank you for this observation, all numbers below ten have now been changed through the manuscript.

Line 509: Ton That Huu and colleagues (2021) does not appear to be in the reference list. Is this referring to Dat et al (2021) because it is not immediately clear if so, as the reference only provides the initials TTH. Please change to Dat et al (2021).

RESPONSE - Thank you, this has been changed to Dat and colleagues (2021).

Line 543: Is this referring to Dat et al 2021 here? Please correct so it matches the reference as it is given.

RESPONSE - Yes, thank you this reference has been amended (Dat et al., 2021)

Line 708: Hesketh-Best et al 2023 is not cited within the manuscript, please remove this reference or cite the reference in the appropriate place.

RESPONSE - Thank you this has now been added to the reference list.

Figures 3 and 5: The media with the 30% (or 3.3%) is in a different order to the media without the 30% (or 3.3%). Specifically, ISP2 30% should come before OAT 30% to keep it consistent.

RESPONSE - Thank you, Figures 3 and 5 have both been changed for continuity as suggested.

Figures 3 and 5: Why was SCA 30% not tested when all other media has a 30% variant included?

RESPONSE - This may appear to be an oversight, but work was not conducted with 30%SCA largely for logistical reasons. Whilst it may have been beneficial to carry out these additional experiments, data for the other media indicate that diluting them did not lead to substantial benefits.

Figure 6: I printed out this figure and the differences in the shades between the same colour is not very prominent. Could they be made a little more distinct?

RESPONSE - Thank you, this figure has since been updated to have a different and more defined set of colours.

Table 2: The activity on different media at pH5 and pH7 can been seen, but at pH7 I do not see the various media. Were the isolates on the pH7 media active or not?

RESPONSE - This is because the strains were isolated at pH7 on their corresponding media, and therefore pH7 at these media types was not tested, this experiment was to look at activity in acid vs. alkali and not pH neutral. Within the OSMAC, pH7 was used with minimal media and changing carbon and nitrogen sources only to assess whether these sources could also trigger activity.

We hope that all comments have now been addressed to the satisfaction of the editor and reviewers.

**Reviews of manuscript FEMSMC-2025-015.R1**

**Reviewer 1**

**Recommendation: Minor Revision**

It was a pleasure to re-review this manuscript. The authors addressed all comments fully, and the manuscript is more scientifically robust and the methods clearer. Figure 7 is now significantly more informative. Overall, I have a few minor corrections listed below, but I would be happy to recommend it once they’ve been addressed.

144 – Please add a sentence to refer to the methodological flow chart (Supplementary Figure 1) at the end of this methods paragraph.

369 – Figure 3: B is missing a bracket ‘B)’

425 – Need a hyphen between Gram positive

448 – 445 – Actinobacteria is a phylum, not a genus. Ensure taxonomic rank is accurate, please

457 - Figure 7 – Significantly improved visualisation. There appears to be an issue however, as the Streptomyces sp. YH4 clusters with the bacillus species (Firmicutes) rather than the Streptomyces pratensis and other Actinobacteria? Is this an issue with the gene sequence or the NCBI taxonomy

527 – Should read ‘to target Actinobacteria, a phylum known for wide bioactivity capabilities,’ phyla is plural and phylum is not capitalised unless used as part of a formal taxonomic name (e.g., Phylum Actinobacteria).

544 – (e.g. Actinobacteria or Bacillus.) aren’t species-level taxonomy. Maybe just remove the bracketed part of the sentence

**Reviewer 2**

**Recommendation: Accept**

All queries and questions have been addressed. I have no further issues.

**Associate Editor Recommendation: Minor Revision**

**EIC Decision: Minor Revision**

**Author's Response to Decision Letter for (FEMSMC-2025-015.R1)**

We thank the reviewers for taking the time to review our revised manuscript. We have attended to all requested further corrections. Regarding the point raised about Figure 7 - we thank the reviewer for bringing the issue to our attention. We investigated the sequences used for the apparently erroneous Streptomyces strain and, strangely, there were no issues identified. However, when taking the complete dataset again and re-drawing the tree, the strain was more accurately placed. This is reflected in the revised version of Figure 7.

Thank you for handling our manuscript, which we now trust is acceptable for publication.

**Reviews of manuscript FEMSMC-2025-015.R2**

**Associate Editor Recommendation: Accept**

**EIC Decision: Accept**
